# Practice Patterns of Spatially Fractionated Radiation Therapy: A Clinical Practice Survey

**DOI:** 10.1016/j.adro.2023.101308

**Published:** 2023-07-09

**Authors:** Nina A. Mayr, Majid Mohiuddin, James W. Snider, Hualin Zhang, Robert J. Griffin, Beatriz E. Amendola, Daniel S. Hippe, Naipy C. Perez, Xiaodong Wu, Simon S. Lo, William F. Regine, Charles B. Simone

**Affiliations:** a College of Human Medicine, Michigan State University, East Lansing, Michigan; b Radiation Oncology Consultants and Northwestern Proton Center, Warrenville, Illinois; c Radiation Oncology, South Florida Proton Therapy Institute, Delray Beach, Florida; d Department of Radiation Oncology, University of Southern California, Los Angeles, California; e Department of Radiation Oncology, University of Arkansas for Medical Sciences, Little Rock, Arkansas; f Innovative Cancer Institute, Miami, Florida; g Clinical Research Division, Fred Hutchinson Cancer Center, Seattle, Washington; h Executive Medical Physics Associates, Miami, Florida; i Department of Radiation Oncology, University of Washington School of Medicine, Seattle, Washington; j Department of Radiation Oncology, University of Maryland School of Medicine, Baltimore, Maryland; k Department of Radiation Oncology, New York Proton Center, New York, New York

## Abstract

**Purpose:**

Spatially fractionated radiation therapy (SFRT) is increasingly used for bulky advanced tumors, but specifics of clinical SFRT practice remain elusive. This study aimed to determine practice patterns of GRID and Lattice radiation therapy (LRT)-based SFRT.

**Methods and Materials:**

A survey was designed to identify radiation oncologists’ practice patterns of patient selection for SFRT, dosing/planning, dosimetric parameter use, SFRT platforms/techniques, combinations of SFRT with conventional external beam radiation therapy (cERT) and multimodality therapies, and physicists’ technical implementation, delivery, and quality procedures. Data were summarized using descriptive statistics. Group comparisons were analyzed with permutation tests.

**Results:**

The majority of practicing radiation oncologists (United States, 100%; global, 72.7%) considered SFRT an accepted standard-of-care radiation therapy option for bulky/advanced tumors. Treatment of metastases/recurrences and nonmetastatic primary tumors, predominantly head and neck, lung cancer and sarcoma, was commonly practiced. In palliative SFRT, regimens of 15 to 18 Gy/1 fraction predominated (51.3%), and in curative-intent treatment of nonmetastatic tumors, 15 Gy/1 fraction (28.0%) and fractionated SFRT (24.0%) were most common. SFRT was combined with cERT commonly but not always in palliative (78.6%) and curative-intent (85.7%) treatment. SFRT–cERT time sequencing and cERT dose adjustments were variable. In curative-intent treatment, concurrent chemotherapy and immunotherapy were found acceptable by 54.5% and 28.6%, respectively. Use of SFRT dosimetric parameters was highly variable and differed between GRID and LRT. SFRT heterogeneity dosimetric parameters were more commonly used (*P* = .008) and more commonly thought to influence local control (peak dose, *P* = .008) in LRT than in GRID therapy.

**Conclusions:**

SFRT has already evolved as a clinical practice pattern for advanced/bulky tumors. Major treatment approaches are consistent and follow the literature, but SFRT–cERT combination/sequencing and clinical utilization of dosimetric parameters are variable. These areas may benefit from targeted education and standardization, and knowledge gaps may be filled by incorporating identified inconsistencies into future clinical research.

## Introduction

Spatially fractionated radiation therapy (SFRT) delivers profoundly heterogeneous doses to the tumor—a major departure from familiar radiation oncology concepts.^1^ Clinical series of SFRT reported markedly high tumor responses and low toxicity in challenging bulky and advanced tumors.^2^, ^3^, ^4^, ^5^, ^6^, ^7^, ^8^, ^9^, ^10^, ^11^, ^12^, ^13^ These enhanced tumoricidal effects are thought to be rooted in SFRT's inherent intratumoral dose heterogeneity^1^^,^^14^ and the ability to deliver large ablative-level fractions to the high-dose domain (“peaks”) of the dose heterogeneity spectrum^14^ while sparing normal tissues and allowing more efficient repair and recovery by virtue of the heterogeneous dosing pattern encountered by organs at risk. Bystander effects in tumor cells adjacent to the high-dose peak regions have been demonstrated in relevant tumor microenvironmental conditions that include increased cytotoxicity over the expected killing by valley dose alone, changes in gene expression, and cytokine production. In addition, preservation of beneficial tumor microenvironment and vasculature within the intratumoral low-dose regions (“valleys”) are postulated to promote crosstalk to improve immune activity, potentially underpinning the observed high clinical tumor responses.^1^^,^^15^^,^^16^

As a result, the clinical interest and use of SFRT has continued to expand in recent years, and both GRID and Lattice radiation therapy (LRT)-based SFRT are now increasingly practiced by radiation oncologists throughout the United States (US) and internationally. However, clinical practice guideline publications for practitioners are just beginning to emerge,^17^^,^^18^ the overall clinical SFRT literature is still sparse,^2^, ^3^, ^4^, ^5^, ^6^, ^7^, ^8^, ^9^^,^^12^^,^^13^ and level I clinical evidence is lacking. It is, therefore, difficult to estimate the current clinical practice pattern in use for SFRT and the rationales and perspectives toward SFRT among radiation oncology practitioners.

Clinical approaches to SFRT, technologies, and techniques have been variable with respect to dose/fractionation schemes and dosimetric planning strategies. Standardization efforts were only recently developed and largely focus on dosimetric parameters.^17^^,^^18^ Two different SFRT platforms, GRID therapy^3^^,^^5^^,^^18^ and LRT,^17^^,^^19^^,^^20^ are preferentially or interchangeably used for various tumor types and sites. GRID therapy is the original form of SFRT, generated by a GRID block (also known as GRID collimator), which forms a heterogeneous dose pattern consisting of a hexagonal pattern of cone-shape pencil beamlets (GRID field).^3^^,^^5^^,^^18^ LRT is a 3-dimensional (3D) form of SFRT with a more variable heterogeneity dose profile.^17^^,^^19^^,^^20^ In aggregate, these clinical, procedural, and technical variabilities likely contribute to inconsistencies in overall clinical practice pattern.

There is an unmet need for a better understanding of how SFRT is currently performed, decided, and implemented; which technologies and planning strategies are deployed for various clinical situations and individual tumors; and how new, unfamiliar SFRT dosimetric parameters are understood and implemented clinically. Such information is important to define practice patterns, to identify disease sites currently treated with SFRT and the practitioners’ pertinent therapeutic rationale, and to gain critical understanding of whether the recently developed standardization guidelines^17^^,^^18^ are actually practiced. Documentation of the SFRT practice pattern is also crucial to identify possible educational needs and knowledge gaps and to inform the focus of future education and research efforts that may serve to improve practice and establish best practices in SFRT.

The purpose of this study was to assess the practice patterns in use for SFRT with GRID and LRT, define prevalence of approaches, and determine practice variations. Specifically, we sought to determine clinical practitioners’ decision making for SFRT indications, choice of SFRT technology, combinations with systemic therapies, radiation therapy planning practice, reporting and quality assurance (QA) processes, practitioners’ use of dosimetric parameters, and their observations of the parameters’ correlation with tumor control.

## Methods and Materials

This survey study was approved by the institutional review board (IRB) of the University of Washington and by the Research Committee of the Radiosurgery Society (RSS), who administered the survey. RSS is a premier national subspecialty radiation oncology society for stereotactic radiation therapy/radiosurgery and targeted radiation therapy using advanced delivery technologies.

### Survey development

The survey was designed by a multidisciplinary group of 10 SFRT experts. Survey questions were developed based on treatment recommendations in the SFRT literature,^2^, ^3^, ^4^, ^5^, ^6^, ^7^, ^8^, ^9^, ^10^, ^11^, ^12^, ^13^ recent publications on SFRT standardization,^17^^,^^18^ and the experts’ clinical experience. Questions were further informed by current controversies in the field, which were incorporated into the answer choices, to elucidate practice patterns in areas of ongoing debate.

### Survey target population

The survey was addressed to radiation oncologists and physicists as the 2 major target groups, and input from basic scientists was also solicited. Recipients included the membership of the RSS GRID, Lattice, Microbeam, and FLASH (GLMF) Radiotherapy Working Groups which consist of a 106-member diverse multidisciplinary group dedicated to the study and clinical practice of GLMF. In addition, to broaden the perspectives beyond the confines of the GLMF working groups, the active RSS membership of radiation oncologists and radiation oncology-related professions was also included.

Questions were tailored (using advanced branching logic methodology) to radiation oncologists and physicists with respect to whether or not they practiced SFRT. Questions were further branched according to whether responders used GRID therapy or LRT because of the fundamental differences in planning and implementation processes between these 2 major SFRT technologies.

### Survey questions: Content

Questions to practitioners included the full spectrum of practice patterns, comprising SFRT indications/patient selection, dose prescription, target volume, combinations with conventional external beam radiation therapy (cERT), and combinations with chemotherapy and immunotherapy. As a measure of whether SFRT was considered by practitioners as an experimental treatment (vs a standard-of-care option), radiation oncologists were asked whether they required or obtained IRB or ethics board approval to treat their patients with SFRT. GRID and LRT technologies and technology platforms were assessed. The use and understanding of new SFRT heterogeneity dosimetric parameters and their attributed influence on tumor control were queried. Attitudes toward biology-based dosimetric parameters, such as equivalent uniform dose (EUD), were examined to gain an understanding of radiation oncologists’ and physicists’ propensity to embrace the “new biology” of heterogenous radiation. Physicists received questions specific to technology platforms, techniques, planning/dosimetry, commissioning, and QA practice. The content of individual survey questions with detailed information on the respective recipient groups are presented in Appendix E1.

Practice type and location, formal training, and clinical patient experience in SFRT were queried. Research activities were estimated through self-reported scientific publication/presentation record of clinical outcome results.

A separate section of the survey addressed radiation oncologists’ practice pattern for 4 primary disease sites that have been most commonly treated with SFRT to date, including head and neck (H&N) cancer, lung cancer, sarcoma, and cervical cancer. Questions were categorized as shown in Tables 1 to 3 and Appendix E1 (sections L through O).

**Table 1:** Practice pattern of patient selection and treatment approach for significance of SFRT in H&N cancer

Selection or planning criterion	Used by percentage of responders
Patient selection	
Treatment of the primary tumor with SFRT	73.3%
Treatment of the bulky lymph nodes with SFRT	100.0%
Treatment of patients with recurrence after prior radiation	60.0%
Change in SFRT dose for patients with recurrence after prior radiation*	13.3%
Treatment of patients with carotid invasion	13.3%
SFRT technology	
Use of GRID	53.3%
Use of Lattice	53.3%
Dose prescription and planning	
Prescription dose†	
15 Gy/1 fraction	29.4%
18 Gy/1 fraction	29.4%
20 Gy/1 fraction	17.6%
GTV as the SFRT target	53.3%
GTV plus a margin as the SFRT target	6.7%
Reduction of the GTV for the SFRT to exclude normal tissues	40.0%
Change in beam angles to maximize sparing of normal tissue	66.7%
Exclusion of spinal cord/brain stem from SFRT target volume	66.7%
Exclusion of mandible from SFRT target volume	60.0%
Reduction of the prescription dose of the conventional external beam radiation therapy because of the SFRT dose	40.0%
Reduction of the normal tissue dose limits for conventional external radiation therapy because of the SFRT dose	13.3%
Use of adaptive therapy replanning for tumor volume changes	33.3%
Combination with systemic therapies	
Allowing chemotherapy or targeted therapy on the day(s) of SFRT	20.0%
Combination of immunotherapy with SFRT sequentially with the radiation therapy course	46.7%
Combination of immunotherapy with SFRT concurrently with the radiation therapy course	33.3%

*Abbreviations:* GTV, gross tumor volume; H&N = head and neck; SFRT = spatially fractionated radiation therapy.

⁎ This question included a request to specify dose in a comment. Single-fraction dose of 8 Gy and 10 Gy was used by 1 responder each, an unspecified dose of <15 Gy was considered (for recurrent tumors) by 1 responder, and 3- to 5-fraction regimes by 2 responders.

† The 3 most common prescription doses are shown. Doses of ≤10 Gy and fractionated schedules in 11.8% each. Questions were tailored according to recommendations in the head and neck SFRT experience and literature and according to areas of uncertainty or controversy. Time gaps between SFRT and conventional external radiation therapy are illustrated in Appendix E1.

**Table 2:** Practice pattern of patient selection and treatment approach for SFRT in lung cancer

Selection or planning criterion	Used by percentage of responders
Patient selection	
Treat stage IIIC (T3-4, N3 M0) lung cancer with SFRT	70.0%
Treat stage IIIA and IIIB lung cancer with SFRT	50.0%
Treat patients with recurrent lung cancer after prior radiation	70.0%
Change the SFRT dose for patients with recurrence after prior radiation*	30.0%
Treat patients who had neoadjuvant chemotherapy	10.0%
Treat the primary tumor only with SFRT	30.0%
Treat both primary tumor and lymph nodes with SFRT	50.0%
SFRT technology	
Use GRID	40.0%
Use Lattice	60.0%
Dose prescription and planning	
Prescription dose†	
18 Gy/1 fraction	42.9%
15 Gy/1 fraction	28.6%
24-30 Gy/3 fractions	21.4%
Use the GTV as the SFRT target	60.0%
Use the GTV plus a margin as the SFRT target	10.0%
Reduce the GTV (for the SFRT) accounting for tumor motion to exclude normal tissues	30.0%
Exclude the esophagus from the SFRT target volume	80.0%
Exclude the brachioplexus from the SFRT target volume	70.0%
Reduce the prescription dose of the conventional external beam radiation therapy because of the SFRT dose	0.0%
Use motion management (eg, abdominal compression, gating) for SFRT	70.0%
Use adaptive therapy replanning for tumor volume changes	60.0%
Combination with systemic therapies	
Allow chemotherapy on the day(s) of the SFRT	20.0%
Combine immunotherapy with SFRT sequentially with the radiation therapy course	50.0%
Combine immunotherapy with SFRT concurrently with the radiation therapy course	20.0%

*Abbreviations:* GTV, gross tumor volume; SFRT = spatially fractionated radiation therapy.

⁎ This question included a request to specify dose in a comment. One responder used 24 Gy/3 fractions, and reduction to 12 to 15 Gy in a single fraction.

† The 3 most common prescription doses are shown. A dose of 20 Gy/2 fractions and higher-dose fractionated schedules were used in 7.1% each. Questions were tailored according to recommendations in the SFRT experience and literature for lung cancer and according to areas of uncertainty or controversy.

**Table 3:** Practice pattern of patient selection and treatment approach for SFRT in sarcoma

Selection or planning criterion	Used by percentage of responders
Patient selection	
Treat soft tissue sarcoma of the trunk/chest	88.9%
Treat soft tissue sarcoma of the extremities	100.0%
Treat soft tissue sarcoma of the retroperitoneum	77.8%
Treat osteosarcoma	22.2%
Treat recurrent tumors after prior radiation	66.7%
Use a different SFRT dose (from the previous question) if you treat patients with recurrence after prior radiation	0.0%
SFRT technology	
Use GRID	44.4%
Use Lattice	44.4%
Prescription dose*	
18 Gy/1 fraction	36.4%
15 Gy/1 fraction	18.2%
20 Gy/1 fraction	18.2%
30 Gy/3 fractions	18.2%
Treat tumors preoperatively	55.6%
After preoperative radiation, give a postoperative boost	11.1%
After preoperative radiation, give a postoperative boost for involved margins only	0.0%
Set a limit to the skin dose from SFRT	33.3%
Exclude brachial plexus from SFRT target volume	77.8%
Reduce the normal tissue dose limits (for conventional external radiation therapy) because of the SFRT dose	22.2%
Combination with systemic therapies	
Allow concurrent chemotherapy	22.2%
Allow chemotherapy on the day(s) of SFRT	22.2%
Combine immunotherapy with SFRT sequentially with the radiation therapy course	22.2%
Combine immunotherapy with SFRT concurrently with the radiation therapy course	22.2%

*Abbreviation:* SFRT = spatially fractionated radiation therapy.

⁎ The 4 most common prescription doses are shown. A low single-fraction dose of 10 to 12 Gy was used in 9.1%. Questions were tailored according to recommendations in the SFRT experience and literature in sarcoma, and according to areas of uncertainty or controversy.

### Survey questions: Format

Question types included multiple-choice questions (1 answer option only) and checkbox questions (multiple answer options, indicated by the prompt “Check all that apply”) for areas of overlapping options (eg, technology platforms or techniques). Preferences were asked using matrix questions which classified the responders’ ratings of how frequently they used specific processes into scales of 1 to 5, for example, whether a certain practice was used always, frequently, sometimes, rarely, or never (eg, Appendix E1, section I). Questions generally invited additional free-text comments using the prompts “Other, please comment” or “Other, please specify” to collect answers not offered in the answer options.

The survey contained a total of 60 questions with an estimated completion time of 10 minutes; completion time varied depending on whether the responder was a radiation oncologist (receiving 46 questions), physicist (19 questions), nonpractitioner, or radiation biologist/molecular biologist (8 questions). The Momentive (San Mateo, CA) platform was used to administer the survey.

### Survey implementation and response

The survey was distributed by RSS through an e-mail containing the survey link and followed by 2 reminder e-mails containing the link. The survey was open from November 9 to December 3, 2021. The survey was anonymous, and the investigators had no access to the RSS’ electronic mailing list or the identity of the recipients. To mitigate nonresponse and conformity bias, the anonymity of the survey was clearly stated in the cover e-mail. A total of 73 responses were received representing a response rate of 8.4% (51/604) for radiation oncologists, 4.8% (17/355) for physicists, and 41.7% (5/12) for radiobiologists/molecular biologists of the combined GLMF Working Group members and the RSS membership.

### Statistical considerations

Recoding of free-text comments was performed before statistical analysis if free-text comments were consistent with one of the question's answer options. Responses were summarized using descriptive statistics including counts, percentages, and means as appropriate. Ratings of dosimetric parameters for clinical use and for influencing local control were compared between GRID and LRT using permutation tests based on 10,000 permutations.^21^ A permutation test is a type of nonparametric test which does not assume the form of the distribution of the data under the null hypothesis, but instead the null distribution is generated by randomly permuting group labels a large number of times. The permutation tests were clustered by respondent to account for nonindependence of multiple ratings from the same respondent, as some respondents rated parameters for both GRID and LRT (paired ratings). Statistical testing was performed in R version 4.0.3 (R Foundation for Statistical Computing, Vienna, Austria). All *P* values were 2-sided, and statistical significance was defined as *P* < .05.

## Results

Over half of the radiation oncologist responders (27/51, 52.9%) currently practiced SFRT, and another 6 (11.8%) planned to begin an SFRT program within 1 to 2 years from the date of the survey. Among the physicists, 12 of 17 (70.6%) practiced SFRT.

### Overall SFRT practice

To estimate radiation oncologists’ judgment of whether SFRT was considered standard-of-care versus investigational/experimental, we asked whether they treated patients with SFRT on an IRB or ethics board-approved research protocol or used SFRT as a standard of care. The vast majority (16/22, 72.7% overall; among 21/22 responders who also provided demographic information, all 11 US and 5/10 international radiation oncologists) delivered SFRT without IRB approval (Appendix E1, section F).

**Figure 1:**
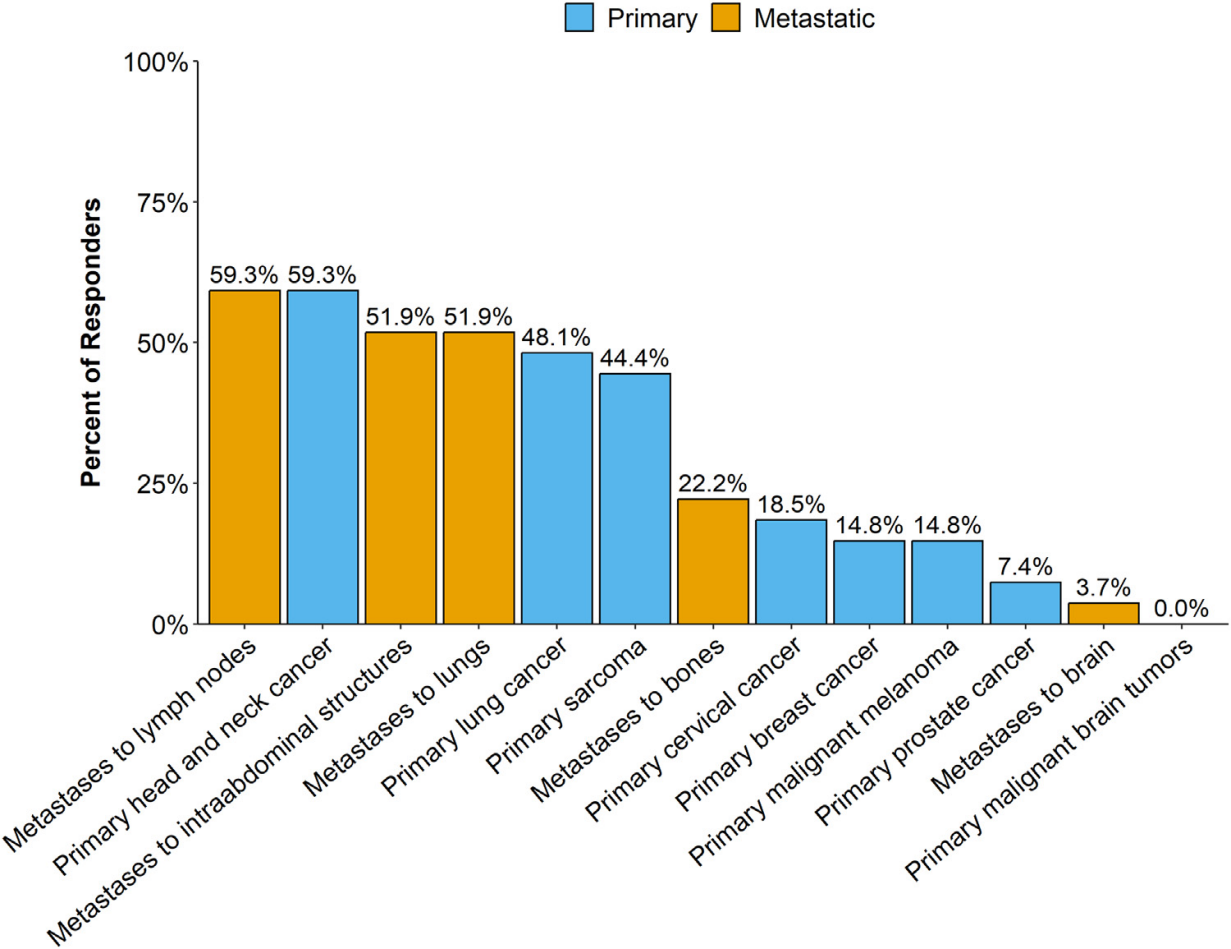
Distribution of disease sites treated with spatially fractionated radiation therapy (SFRT). Proportion of radiation oncologists using SFRT for specific tumor types and tumor sites are presented. Metastatic tumor sites (“Metastases to…”), treated palliatively, are shown in orange bars, and advanced bulky (nonmetastatic) primary tumors (“Primary…”) treated definitively with curative intent in blue columns. Data are sorted by frequency. Data show the answer to question “What type of tumors do you treat with SFRT? (Check all that apply).” All 27 radiation oncologists who practice SFRT answered this question (107 answers). Responders could choose more than 1 answer option.

The distribution of tumor types and sites is shown in Fig. 1. Among palliative indications, metastases to lymph nodes, intra-abdominal structures, and lung were most common and treated by over half of responders. Among the advanced/bulky primary tumor sites treated with curative intent, H&N, lung cancer, and sarcoma prevailed. Primary breast cancer was treated by 14.8%. SFRT was not given to primary intracranial tumors and rarely given to brain metastases (3.7%).

### Major treatment concepts and specific radiation therapy approaches

#### SFRT dose/fractionation and sequencing with cERT

**Figure 2:**
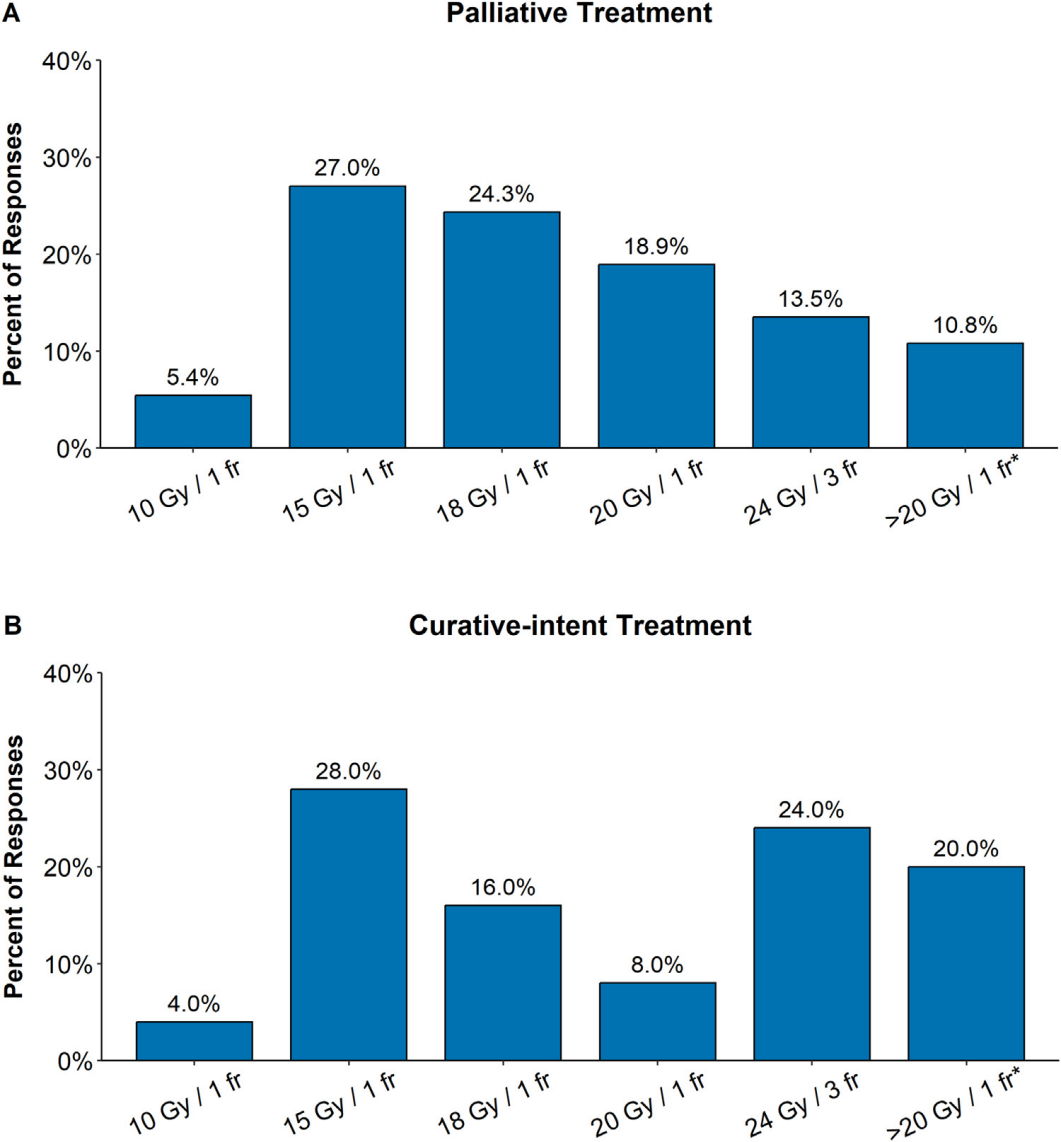
Spatially fractionated radiation therapy (SFRT) dose used for palliative and curative-intent treatment. Distribution of SFRT dose schedules for (A) palliative and (B) curative-intent treatment is shown as percentage of total responses. The data show predominance of 15 Gy to 18 Gy single-fraction treatments and a trend toward higher-dose single factions of 20 Gy in palliative treatment. In curative-intent treatment 15-Gy single fractions and fractionated treatment (24 Gy/3 fractions) are most common, while 20-Gy single fractions are rare. Data show the answer to questions, “What general SFRT dose schedules do you use for palliatively treated patients? (Check all that apply)” and “What general SFRT dose schedules do you use for curatively treated patients? (Check all that apply).” Among the 27 SFRT-practicing radiation oncologists, 26 answered the question for palliative (37 responses), and 21 answered the question for curative treatment (25 responses). Responders could choose more than 1 answer option. *The column labeled “>20 Gy/ 1 fr” contains miscellaneous schedules of a higher dose, most commonly in 3 to 5 fractions (Appendix E1, section C).

For palliation, a single SFRT fraction of 15 or 18 Gy was the most common schedule (51.3% of responses; Fig. 2A), followed by 20 Gy in 1 fraction (18.9%). Fractionated regimens including 24 Gy in 3 fractions (13.5%) and 3- to 5-fraction regimens of variable dose were also used.

In the curative-intent treatment of primary malignancies, a lower fraction size of 15 Gy in 1 fraction (28.0%) and fractionated regimens of 24 Gy in 3 fractions (24.0%) prevailed (Fig. 2B). Dose/fractionation schedules varied for the 4 individually assessed primary malignancies with single fractions of 15 Gy to 18 Gy predominating in H&N, lung cancer, and sarcoma, and 3-fraction schedules more common in cervical cancer. Results are summarized in Tables 1 to 3 and Appendix E1, section N. Lower-dose regimens (10 Gy in 1 fraction) were uncommon (≤5%) in both palliative and curative-intent treatment (Fig. 2).

The recommended practice of combining SFRT with cERT^3^^,^^4^^,^^7^^,^^9^, ^10^, ^11^, ^12^, ^13^ was common in palliative settings (78.6%) and highly prevailed in curative-intent treatment (85.7%). The majority (50.0% and 57.1% of responses) delivered SFRT before cERT, respectively. However, alternative sequencing was employed by 28.6% of practitioners, using interdigitated schedules (4/21, 19.0%) and SFRT after cERT (2/21, 9.5%) in curative-intent treatment and in similar proportions in palliative SFRT (5/28 [17.9%] and 3/28 [10.7%], respectively). In curative-intent treatment, a substantial proportion (4/21, 19.0%) reduced the cERT dose (from the dose standard in definitive conventional radiation) because of the SFRT dose contribution. This cERT dose reduction was particularly seen in H&N cancer (40.0%; Table 1).

#### Combination of systemic therapy with SFRT

In palliatively treated tumors, 37.5% of practitioners were willing to combine SFRT with chemotherapy concurrently with the cERT component of the treatment course. Only 12.5% allowed chemotherapy to be given on the day(s) of the SFRT fraction(s). In curatively treated tumors, the proportion of concurrent chemotherapy trended higher (45.5%), but only 9.1% permitted chemotherapy during the SFRT fraction(s), and this was consistent across the 4 specific primary disease sites (Table 1, Table 2, Table 3, Appendix E1, section N).

For palliation, the overall practice of combining immunotherapy with SFRT-containing regimens (35% of responders) was similar in frequency to that of combining chemotherapy. However, a higher proportion (25.0%) permitted immunotherapy during the SFRT fraction(s), whereas only 10.0% limited the combination of immunotherapy to the cERT portion of the course.

In curatively treated tumors, the propensity to combine immunotherapy was overall lower (28.6%) than for the combination with chemotherapy (45.5%), but most of those allowing this combination administered the immunotherapy also during the SFRT fraction(s) (66.7% of those permitting combined immunotherapy). Disease-specific approaches to immunotherapy in primary tumors are presented in Tables 1 to 3 and Appendix E1, section N.

### Technology and technical implementation

#### SFRT technique and platform

Radiation oncologists most commonly practiced GRID therapy (50.0% of responses), either GRID collimator-based (22.5%) or multileaf collimator-based (27.5%), closely followed in frequency by LRT (45.0%). Both GRID and LRT were practiced by 19.0% of responders (4/21). The use of linear accelerators prevailed, with overlapping use of GRID collimators (16.1%), multileaf collimator (17.9%) for GRID therapy, and volumetric arc therapy (25.0%) and intensity modulate radiation therapy (17.9%) for LRT. CyberKnife (8.9%), TomoTherapy (5.4%), and proton therapy (5.4%) were also used, but no carbon ion-based SFRT was reported. Physicists’ assessments overall matched that of the radiation oncologists (Appendix E1, sections E and K). Use of the full range of commercially available planning and QA systems were reported, and systems overlapped widely (Appendix E1, section K). No in-house developed planning software was reported.

### Dose prescription and planning

In GRID therapy, the dose was predominantly prescribed to the 3D gross tumor volume (GTV) without an additional margin (62.5%) and to maximum dose (37.5%) but not to the GTV with a margin or to a defined prescription point at tumor depth. In LRT, the dose was most commonly prescribed to the vertex tumor volume (volume of the vertices; ie, the GTV minus an inward margin) (44.4%), to the GTV without a margin (27.8%), and, rarely, to the GTV with an additional margin (5.6%). Preferences on prescription volumes, organs-at-risk management, and treatment planning differed in the individual disease sites and are described in Tables 1 to 3 and Appendix E1, section N.

#### Clinical use of SFRT dosimetric parameters and attributed clinical significance

**Figure 3:**
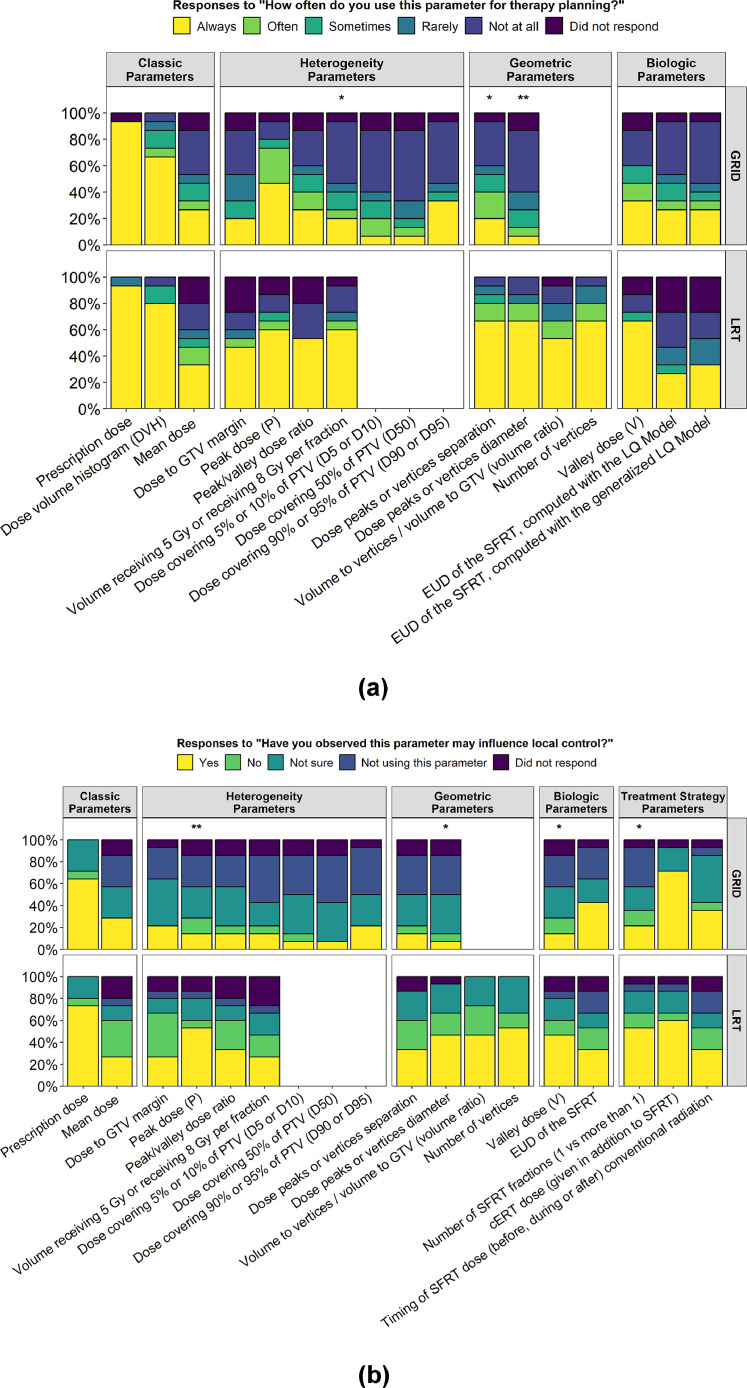
Usage and assigned predictive significance of spatially fractionated radiation therapy (SFRT) dosimetric parameters in GRID therapy compared with Lattice radiation therapy (LRT). (A) The distribution of how commonly each individual dosimetric parameter is clinically used, comparing GRID and LRT practitioners (yellow = always used; light green = often; dark green = sometimes; aqua = rarely; blue = not at all, purple = no answer^†^). Upper panel = GRID therapy practitioners (n = 15), lower panel = LRT practitioners (n = 15). Statistically significant differences among dosimetric parameter use between GRID and LRT are denoted as *P < .05 for the difference in “always used” responses between GRID and LRT practitioners and ^⁎⁎^*P* < .01 for the difference in “always used” responses between GRID and LRT practitioners. Parameters that are comparable between GRID and LRT include: classic parameters (GRID prescription dose, dose-volume histogram, and mean dose), heterogeneity parameters (peak dose, valley/peak dose ratio or peak/valley dose ratio, dose to gross tumor volume [GTV] margin, and volume receiving 5 Gy or receiving 8 Gy per fraction), geometric parameters (peak width [defined at 50% of the maximal dose] and peak-to-peak distance), and biologic parameters (valley dose, equivalent uniform dose [EUD] of the SFRT computed with the linear quadratic model, and EUD of the SFRT computed with the generalized linear quadratic model). The remainder of the parameters are not comparable between GRID and LRT due to differences in current technique and standards of parameter reporting among the 2 SFRT techniques (no data for these parameters are reported in the graph). Panel A represents the answers to questions, “When using GRID therapy, how commonly do you use the following dosimetric parameters for GRID therapy planning?”; and, “When using Lattice therapy, how commonly do you use the following dosimetric parameters for Lattice therapy planning?” Responses were provided by 24 of the 27 SFRT-practicing radiation oncologists, with 15 for GRID parameters and 15 for LRT parameters (6 respondents rated the use of both GRID and LRT parameters). (B) The clinicians’ assessment regarding the observed influence of the same individual dosimetric parameters (as in A) for local tumor control in their patients, comparing GRID and LRT practitioners (yellow = yes, ie, influence on tumor control was observed; light green = no; dark green = unsure; blue = parameter not used; purple = no answer^†^). Upper panel = GRID therapy practitioners (n = 14), lower panel = LRT practitioners (n = 15). In addition to the parameters presented in panel A, parameters that assesses overall treatment planning strategy, number of SFRT fractions (1 vs more than 1), cERT dose (given in addition to SFRT), and timing of SFRT dose (before, during, or after conventional radiation) were also evaluated. Panel B represents the answers to the questions, “In patients you treat with GRID therapy, have you observed that any of the following parameters may influence local tumor control?” and “In patients you treat with Lattice therapy, have you observed that any of the following parameters may influence local tumor control?” Responses were provided by 23 of the 27 SFRT-practicing radiation oncologists. One responder, who rated the use of dosimetric parameters, did not respond to any questions on the influence of parameters on local control and was excluded for the analysis, leaving 14 GRID practitioners and 15 LRT practitioners (6 rated both GRID and LRT). *P < .05 for the difference in “Yes” responses between GRID and LRT practitioners. ^⁎⁎^*P* < .01 for the difference in “Yes” responses between GRID and LRT practitioners. **^†^**“No answer” indicates no response given for the specific parameter. Respondents who provided an answer to at least 1 parameter among the answer options were included in the calculations. *Abbreviations:* DVH = dose-volume histogram; EUD = equivalent uniform dose; GTV = gross tumor volume; LQ = linear quadratic; LRT = Lattice radiation therapy; PTV = planned total volume; SFRT = spatially fractionated radiation therapy.

GRID and LRT dosimetric parameters that were recently recommended by the pertinent white papers^17^^,^^18^ were tested for the frequency of their clinical use and for practitioners’ clinical observations and perspectives on the potential influence of the dosimetric parameters on tumor control. Figure 3 illustrates the frequency of clinical use of the individual parameters and their clinician-observed influence on local tumor control.

##### Clinical use of SFRT dosimetric parameters

SFRT prescription dose was always used clinically by 93.3% of both GRID and LRT practitioners, and the dose-volume histogram was always used by two-thirds or more of GRID (66.7%) and LRT (80.0%) practitioners. In contrast, the utilization of the new complex heterogeneity dose parameters and geometric parameters showed major differences between GRID and LRT (*P* = .008 and *P* < .001, respectively; Fig. 3A). In GRID therapy, dose to GTV was always used by 46.7% of responders, followed by 33.3% of responders for dose covering 90% or 95% of planned target volume and for valley dose. All other dosimetric parameters were always used by only 6.7-26.7% of responders (Fig. 3A, Appendix E1, section I).

LRT showed a different pattern. Six of the 14 dosimetric parameters were always used by at least two-thirds of responders, and 10 of 14 by at least half of responders (Fig. 3A). In particular, the heterogeneity and geometric SFRT parameters were significantly more commonly used by LRT than by GRID practitioners. These heterogeneity parameters included volume receiving 5 Gy or receiving 8 Gy per fraction (always used: 60.0% by LRT practitioners vs 20.0% by GRID practitioners; *P* = .027), dose peaks (vertices) separation (66.7% vs 20.0%, respectively; *P* = .013), and dose peaks (vertices) diameter (which define the heterogeneity pattern, 66.7% vs 6.7%, respectively; *P* = .001).

##### Attributed influence of SFRT dosimetric parameters on local control

In GRID therapy, only GRID prescription dose and cERT dose were considered influential for local tumor control by a substantial proportion of practitioners (64.3% and 71.4%, respectively; Fig. 3B). Mixed responses and high degree of uncertainty regarding an influence on local control (“not sure” answer) prevailed for the heterogeneity dose parameters.

In LRT, the LRT prescription dose and cERT dose predominated as parameters with clinician-observed influence on local control (73.3% and 60.0%, respectively), similar to GRID therapy (Fig. 3B). However, a larger proportion of LRT than GRID practitioners reported an observed influence of several heterogeneity and geometric parameters on local control, particularly for peak dose (53.3% vs 14.3%; *P* = .008), valley dose (46.7% vs 14.3%; *P* = .028) and dose peaks or vertices diameter (46.7% vs 7.1%; *P* = .019). The outcome-predictive significance attributed to EUD was similar in LRT and GRID therapy (33.3% vs 42.9%; *P* = .62).

In view of the increased attention regarding the significance of valley dose,^22^^,^^23^ the question of appropriate valley dose was posed to all practitioners. The majority of radiation oncologists (58.3%) considered an as-low-as-achievable valley dose or ≤5 Gy in 1 fraction (20.8%) most appropriate, and few (16.7%) were uncertain of its significance. Results for physicists were similar (55.6%, 0%, and 22.2%, respectively). None in either profession considered valley dose unimportant (Appendix E1, sections K and J).

### Demographic findings, education, and research

#### Practice type and location

Practice in an academic hospital/university (55.9%) or hybrid academic/private practice (8.8%) prevailed among SFRT practitioners (Appendix E1, section P). Although most responders (physicians and physicists) were in the US (61.8%), nearly 2 in 5 practiced outside the US (38.2%), predominantly in Asia (38.5% of international practitioners), Europe (30.8%), and Latin America (30.8%).

#### SFRT training and clinical practice experience

SFRT training was uncommon in residency (GRID 16.7%, LRT 8.3%, proton SFRT 0.0%). Most practitioners (72.7%) reported 9 years or less of SFRT practice, and most were in midcareer (10-19 years after residency/training: 50.0%; Appendix E1, section P).

Most practitioners (40.0%) reported having treated a total of 6 to 35 patients with SFRT (6-20 patients: 26.7%; and 21-35 patients: 13.3%). Twenty percent treated over 75 patients, and none treated over 150 patients (Appendix E1, section B). The majority of physicists had SFRT commissioning (66.7%), and all had patient-specific QA experience or practiced in a center where patient-specific QA is performed for SFRT.

#### Research activities

Forty percent of radiation oncologists reported having published their clinical SFRT outcome data or presented them at scientific meeting(s); 37.5% enrolled or planned to enroll patients into SFRT clinical trials, and 52.0% have access to a radiobiologist or biologist.

## Discussion

Our results suggest that SFRT is already well established in clinical practice. The responders’ common practice (72.7% overall, 100% in the US) to deliver SFRT without IRB or ethics board approval indicates that the vast majority consider SFRT as one of the standard-of-care radiotherapeutic options for advanced bulky tumors. Our observed expansion of SFRT indications from the more established palliative treatment to increasingly curative-intent treatment of nonmetastatic advanced primary malignancies (Fig. 1), which has been only recently developed,^5^, ^6^, ^7^, ^8^, ^9^, ^10^, ^11^, ^12^, ^13^ suggests that SFRT practice is in a state of growth. These results are in agreement with frequent requests for education and research in SFRT observed by the RSS GLMF Radiotherapy Working Groups.

### Major treatment concepts and specific treatment approaches

Our results show that major therapeutic approaches are largely consistent and in accordance with the SFRT literature.^2^, ^3^, ^4^, ^5^, ^6^, ^7^, ^8^, ^9^, ^10^, ^11^, ^12^, ^13^ High ablative-size fractions were generally used for SFRT, prescription doses followed the major publications,^2^, ^3^, ^4^, ^5^, ^6^, ^7^, ^8^, ^9^^,^^12^^,^^13^ and a trend to higher fraction sizes was seen in palliative treatment of metastases/recurrences relative to curative-intent treatment of primary malignancies (Fig. 2). When querying the practice of dose schedules, the SFRT prescription doses were generally in accordance with the literature. Chemotherapy was combined with SFRT/cERT regimens for curative-intent treatment by most responders per the standards of care for systemic therapy in specific primary tumors (Tables 1-3; Appendix E1, section N).

Variability in practice, however, emerged within the specific approaches to the combination of SFRT with cERT and the sequencing of SFRT with cERT. Although, as recommended,^3^, ^4^, ^5^, ^6^, ^7^^,^^9^, ^10^, ^11^, ^12^, ^13^ SFRT was generally combined with cERT for most palliative (78.6%) and definitively treated tumors (85.7%), just under 15% did not add cERT to the SFRT for curative-intent treatment of primary malignancies, in which the addition of cERT is particularly recommended.^6^^,^^7^^,^^9^, ^10^, ^11^, ^12^, ^13^ Sequencing of SFRT with cERT showed major variability in both palliative and, surprisingly, curative-intent treatment. Our observation that only half of responders administered SFRT before the cERT and over one-fourth practiced SFRT interdigitated with or after cERT was unexpected, particularly for curative-intent treatment. This is only partially explained by uncertainties in the current literature, considering that the pre-cERT delivery of SFRT has emerged as the major approach^5^, ^6^, ^7^^,^^9^, ^10^, ^11^, ^12^ and may be advantageous for potential immunogenic effects or synergy with subsequent immunotherapy due to priming effect from heterogeneous dosing.^16^^,^^24^^,^^25^

Furthermore, for the necessary combination of SFRT with cERT in curative-intent treatment, almost 1 in 5 practitioners (19%) reduced the cERT dose in the combined SFRT/cERT regimen, predominately in primary H&N cancers (Table 1), despite contrary literature recommendations.^6^^,^^7^^,^^13^ These practice variabilities underscore the need for additional education in the field of SFRT and the importance to standardize clinical treatment approaches in institutional settings and for prospective studies.

### SFRT practice in the context of multimodality systemic therapy

The practice of chemotherapy with SFRT-containing radiation therapy regimens showed more consistency was well accepted among responders and was concordant with the literature.^6^^,^^7^^,^^9^^,^^10^^,^^13^ Concurrent chemotherapy was largely administered during the cERT portion of treatment, with only a minority of responders (≤12.5%) giving chemotherapy during the SFRT fraction(s) for both palliative and curative-intent treatment. This reluctance by practitioners may reflect a growing awareness and consideration in the radiation oncology community that administration of chemotherapy concurrently with high-dose fraction regimens could potentially destroy beneficial immune cells or disrupt a positive immune microenvironment.^26^

Immunotherapy combinations with SFRT, which are overall largely unexplored in the literature, were practiced more commonly than expected, by approximately one-third of clinicians (35.0% in palliative and 28.5% in curative-intent treatment). Surprisingly, immunotherapy was administered during the SFRT fraction(s) more commonly, by 25.0% and 19.0%, respectively. This observation is particularly unexpected in the curative-intent setting, as the only report of combined immunotherapy with SFRT is in palliative treatment.^27^ This already-existing practice of combining immunotherapy with SFRT may provide an opportunity to extract outcome data from this practice that may help elucidate response and toxicity of immunotherapy/SFRT combinations. Practitioners’ willingness to combine immunotherapy is also encouraging for the feasibility of future clinical trials aiming to investigate such combinations.

### Technology and technical implementation

As expected, GRID therapy, which was developed much earlier^2^, ^3^, ^4^, ^5^^,^^11^ than LRT,^8^^,^^9^^,^^19^ was the most common SFRT technology, although it was closely followed by LRT. Although linear accelerator platforms predominated, the utilization of a wide range of other platforms suggests the adaptability of SFRT to many delivery technologies. However, this technological variability will require close attention to resultant dosimetric variability, especially for clinical trials. The substantial proportion of proton therapy in GRID (but not LRT) attests to the increasing interest in proton-based GRID as an area of ongoing exploration for its potential of improved dose profiles.^28^

### Clinical use and attributed clinical significance of SFRT dosimetric parameters

New heterogeneity dosimetric parameters have been an area of lacking standardization in SFRT that has only been recently addressed by major guideline initiatives,^17^^,^^18^ and their actual utilization of these guidelines in clinical practice has remained unclear.

Our data indicate major practice variations in the utilization of currently recommended heterogeneity dose parameters in SFRT, a just-emerging use of these parameters in GRID, and a wider use in LRT (Fig. 3A). In aggregate, these findings suggest that a fundamental transition in clinical SFRT practice is currently taking place from a uniform-striving paradigm to heterogeneity-based dosimetric concepts, which are inherently intertwined with the putative response mechanisms of SFRT.^25^^,^^29^

However, an understanding of the specific clinical outcome correlations of the new complex heterogeneity dose parameters with clinical treatment response has yet to fully evolve, as is shown by the variability in practitioners’ attributed influence of heterogeneity parameters on local control (based on their clinical practice observations) and the different patterns of variability between GRID and LRT. LRT practitioners more commonly and with greater certainty attributed predictive relevance for local control to the heterogeneity dose parameters than GRID practitioners (Fig. 3B). Although these differences between GRID and LRT practitioners are challenging to explain and may be related in part to the higher clinical use of and higher focus on heterogeneity parameters in LRT (owing to greater inherent interplan variability of LRT heterogeneity dose patterns) than in GRID therapy, the overall variability in attributed outcome correlations points to a major knowledge gap in the field of SFRT. Such gaps can be filled through institutional studies that rigorously collect heterogeneity dose parameters, national database/registry research efforts (which are currently evolving) or prospective clinical trials.

### Biologic-based dosimetric parameters

Contrary to their divergent attitudes toward the clinical outcome significance of the heterogeneity and geometric parameters, EUD was considered significant for local control by at least one-third among both GRID (42.9%) and LRT (33.3%) practitioners (Fig. 3B). This suggests an evolving awareness and acceptance of biologic dosimetric parameters for routine clinical use by a substantial proportion of practitioners. This notion is further supported by clinicians’ attitudes toward valley dose, specifically their preference for low valley doses. Valley dose has recently gained increasing attention through its postulated impact on preservation of immune function and tumor vasculature (within low-dose regions),^23^ thereby defining it as one of the biologic dosimetric parameters rooted in novel biologic principles^25^ of SFRT. Indeed, several have reported that the overall tumor response is most correlated to the valley dose.^22^^,^^23^

It is beyond the scope of this survey to elucidate the specific properties of individual dosimetric parameters for their observed correlation with local control. A survey of clinician-observed outcome correlations is a crude measure of the potential relevance for actual clinical outcomes (compared with the direct response data obtained from a patient cohort). These results should, therefore, be interpreted with caution. Instead, the trends derived from our practitioner-reported clinical observations may suggest potential candidate parameters that might be considered for more detailed evaluation in future clinical investigations of SFRT. Such narrowing of candidate parameters may be important in view of the current dearth of systematic data correlating specific heterogeneity dose parameters with tumor control outcomes in clinical patients. Our findings thus suggest an urgent need for clinical investigations that rigorously incorporate heterogeneity and biologically based parameters. Such research endeavors appear realistically achievable based on the overall motivation of SFRT practitioners, many of whom have published or presented their clinical data (40%) and/or enrolled patients into SFRT clinical trials (37.5%). In the interim, clear guidance to clinicians is needed regarding which dosimetric parameters to generate and report for both clinical care and research investigations.

### Limitations

This practice survey has the inherent limitations of questionnaire-based survey research, such as participation rate, nonresponse, and conformity bias, and the dependence on accurate responses provided by participants. The survey was anonymous to mitigate nonresponse and conformity bias.

Despite implementation of pertinent measures, including detailed survey description during launch, survey reminders, and limited survey length, our sample size of 73 responders is relatively small. This is not entirely unexpected. Although SFRT has undergone major expansion in recent years, the community of practitioners is still small compared with the overall membership of RSS. Although we could have limited the survey to the 106 GLMF Working Group members, which likely constitute the vast majority of responders, thereby achieving a much higher response rate, we chose to also include the much larger RSS membership to broaden the spectrum of viewpoints. This larger denominator, in turn, reduced our response rate. The limitation from the low sample size particularly applies to the assessments of practice patterns in the 4 individual primary malignancies. Although our overall and especially disease-specific findings should be considered preliminary and require confirmation with future studies, this practice survey is, to our knowledge, the only such record to date and serves as a first data set to elucidate clinical practice patterns of SFRT.

## Conclusion

This practice survey suggests that a clinical practice pattern of GRID and Lattice SFRT has already evolved, and that SFRT is considered by practitioners as one of the standard-of-care options for bulky advanced tumors. Major clinical decision-making and SFRT dosing and technique choices are overall consistent and follow the SFRT literature, but SFRT–cERT combination and dosing and the sequencing of the SFRT with cERT show substantial variability of practice. The clinical use of SFRT-specific heterogeneity dosimetric parameters and practitioners’ understanding of their influence on tumor control are highly variable. These identified trends in the current SFRT practice pattern may help define knowledge gaps amenable to targeted education and inform focus areas for standardization efforts and future research.

## Acknowledgment

The authors are grateful to the Radiosurgery Society for their collaboration in this project, without which this research would not have been feasible. Specifically we thank the RSS for reviewing our study proposal and for facilitating the distribution of the survey.

## Disclosures

Nina A. Mayr reports that administrative support (distribution of the survey to recipients) was provided by the Radiosurgery Society. Beatriz E. Amendola reports speaker honorarium. Robert J. Griffin reports speaker honorarium and meeting travel support. Simon Lo reports a relationship with the Radiosurgery Society that includes board membership. Majid Mohiuddin has the patent “Proton Spatially Fractionated Radiotherapy.” Naipy C. Perez reports speaker honorarium. James W. Snider reports consulting fee and speaker honoraria and has the patent “Proton Spatially Fractionated Radiotherapy.” Xiaodong Wu has the patent “Method of 3D Lattice Radiotherapy” and reports speaker honoraria and waived meeting registration fee.

Nina A. Mayr, Charles B. Simone II, Hualin Zhang, Xiaodong Wu, and Robert Griffin report a relationship with Radiosurgery Society that includes committee leadership. Majid Mohiuddin and James W. Snider report a relationship with Radiosurgery Society that includes committee co-leadership.

## Appendix Supplementary materials

SFRT-Pract-Survey_Appendix_2.13.23 Image, application 1

## Footnotes

Supplementary material associated with this article can be found in the online version at doi:10.1016/j.adro.2023.101308.
