# Supplementary material for: Practice Patterns of Spatially Fractionated Radiation Therapy: A Clinical Practice Survey
Source: Adv Radiat Oncol. 2023 Jul 9;9(2):101308. doi: 10.1016/j.adro.2023.101308 (PMC10885580; doi:10.1016/j.adro.2023.101308)
Supplement: SFRT-Pract-Survey_Appendix_2.13.23 [file mmc1.docx]

# **Appendix**

**Current Clinical Practice Patterns of Spatially Fractionated Radiation Therapy:**

**A Clinical Practice Survey**

Note:

Survey questions, answer options of the questions and results are presented in the tables. Additional analyses (e.g. group comparisons) are presented in additional tables.

Questions are grouped according to subject areas (sections A through Q). Questions were tailored to radiation oncologists and physicists with respect to whether or not they practiced SFRT, and further tailored according to whether responders used GRID therapy or Lattice radiation therapy. The target population of the questions is indicated for each section. Question number (e.g. ‘Qu 1’) indicates the question’s appearance in the survey; however, actual question sequence and number seen by the responders varies by target population.

Questions include:

*Multiple choice questions:* one answer option among a fixed group of answer choices.

*Check box questions:* multiple answer options among a fixed group of answer choices.

*Matrix/rating scale questions:* using a rating scale (Likert scale) to rate multiple answer choices, e.g., rate how commonly a process is used).

The question type is indicated for each question.

**A. Use of SFRT and profession**

(Questions to all responders)

**Qu 1: Do you use SFRT (GRID or Lattice therapy) in your practice?**

**Qu 2: What is your profession?**

| **Answer Choices** | **Radiation Oncologists**  Percentage No. | | **Physicists**  Percentage No. | | **Biologist/Scientist**  Percentage No. | | **Total**  No. | |
| --- | --- | --- | --- | --- | --- | --- | --- | --- |
| Yes | 52.9% | 27 | 70.6% | 12 | − | | | 39 |
| No | 35.3% | 18 | 23.5% | 4 | − | | | 22 |
| I plan to start  using SFRT in the next 1-2 years | 11.8% | 6 | 5.9% | 1 | − | | | 7 |
| N/A ^1)^ | − | | − | |  | 5 | | 5 |
| Total | 100% 51 | | 100% 17 | | 100% 5 | | | 73 |

Multiple choice questions. Questions answered by all responders.

Note:

^1)^ Biologist/Scientist did not receive this question.

**B. Overall SFRT practice, disease sites**

(Questions to all radiation oncologists)

**Qu 3: How many patients total (estimated) have you treated with SFRT?**

| **Answer Choices** | **Radiation oncologists**  Percentage No. | | |
| --- | --- | --- | --- |
| None | 16.7% | | 5 |
| <5 | 20.0% | | 6 |
| 6-20 | 26.7% | | 8 |
| 21-35 | 13.3% | | 4 |
| 36-75 | 3.3% | | 1 |
| 76-150 | 20.0% | | 6 |
| >150 | 0% | | 0 |
| Total | 100% | 30 | |

Multiple choice question. Answered by 30 radiation oncologists.

**Qu 4: What type of tumors do you treat with SFRT?  Check all that apply**

| **Answer choices** |  | **Radiation oncologists** | | | | | |  |
| --- | --- | --- | --- | --- | --- | --- | --- | --- |
|  |  | Percentage of responders | | | No. of responses | | | |
| Metastases to lymph nodes | | | | 59.3% | | 16 | | |
| Primary (non-metastatic) head and neck cancer | | | | 59.3% | | 16 | | |
| Metastases to intraabdominal structures | | | | 51.9% | | 14 | | |
| Metastases to lungs | | | | 51.9% | | 14 | | |
| Primary (non-metastatic) lung cancer | | | | 48.2% | | 13 | | |
| Primary (non-metastatic) sarcoma | | | | 44.4% | | 12 | | |
| Metastases to bones | | | | 22.2% | | 6 | | |
| Primary (non-metastatic) cervical cancer | | | | 18.5% | | 5 | | |
| Primary (non-metastatic) breast cancer | | | | 14.8% | | 4 | | |
| Primary (non-metastatic) malignant melanoma | | | | 14.81% | | 4 | | |
| Primary (non-metastatic) prostate cancer | | | | 7.41% | | 2 | | |
| Metastases to brain | | | | 3.70% | | 1 | | |
| Primary malignant brain tumors | | | | 0% | | 0 | | |
| **Total** | | | − | | | | 107 | |

Check box question. Answered by 27 radiation oncologists.

Note:

Data are presented as percentage of radiation oncologists, who treated a specific tumor/tumor site (Percentage of responders). Responders could choose more than one answer option.

One responder also treated primary hepatocellular carcinoma (information based on free-text comments).

**C. SFRT dose/fractionation, sequencing with conventional external beam radiation**

(Questions to all radiation oncologists)

**Qu 5: What general SFRT dose schedules do you use for PALLIATIVELY treated patients? Check all that apply**

| **Answer** **Choices** | **Responses**  Percentage No. | |
| --- | --- | --- |
| 10 Gy in 1 fraction | 5.4% | 2 |
| 15 Gy in 1 fraction | 27.0% | 10 |
| 18 Gy in 1 fraction | 24.3% | 9 |
| 20 Gy in 1 fraction | 18.9% | 7 |
| 24 Gy in 3 fractions | 13.5% | 5 |
| More than 20 Gy in 1 fraction: (Specify) ^1)^ | 10.8% | 4 |
| Total | 99.9% | 37 |

Check box question. Answered by 26 radiation oncologists.

Note:

Percentages are percent of the total number of 37 responses. Responders could choose more than one answer option.

^1)^ Responses to the “More than 20 Gy in 1 fraction” option included three multi-fraction regimens, 30 Gy/3 fractions, 45 Gy/3 factions and 66.67 Gy/5 fractions; and one single-fraction regimen, 10-20 Gy/1 fraction.

**Qu 6: What general SFRT dose schedules do you use for CURATIVELY treated patients with primary (non-metastatic) tumors?  Check all that apply**

| **Answer choices** | **Responses**  Percentage No. | | |
| --- | --- | --- | --- |
| 10 Gy in 1 fraction | | 4.0% | 1 |
| 15 Gy in 1 fraction | | 28.0% | 7 |
| 18 Gy in 1 fraction | | 16.0% | 4 |
| 20 Gy in 1 fraction | | 8.0% | 2 |
| 24 Gy in 3 fractions | | 24.0% | 6 |
| More than 20 Gy in 1 fraction  (Specify) ^1)^ | | 20.0% | 5 |
| Total | | 100% | 25 |

Check box question. Answered by 21 radiation oncologists.

Note:

Percentages are percent of the total number of 25 responses. Responders could choose more than one answer option.

^1)^ Responses to the answer option “More than 20 Gy in 1 fraction” included one multi-fraction regimen, 25 Gy/5 factions; one single-fraction regimen, 10-15 Gy/1 fraction; a dose of 24-28 Gy (fraction number unspecified); and 2 responses without specification.

**Qu 7: Do you combine SFRT with conventionally fractionated external beam radiation (EBRT) for PALLIATIVE treatment?  Check all that apply**

| **Answer choices** | **Responses**  Percentage No. | | |
| --- | --- | --- | --- |
| No, I treat with SFRT alone | 17.9% | 5 |  |
| Yes, SFRT before conventional EBRT | 50.0% | 14 |  |
| Yes, SFRT concurrent (interdigitated) with conventional EBRT | 17.9% | 5 |  |
| Yes, SFRT after conventional EBRT | 10.7% | 3 |  |
| Other (Specify) ^1)^ | 3.6% | 1 |  |
| Total | 100.1% | 28 |  |

Check box question. Answered by 26 radiation oncologists; 3 responders, who indicated they did not use SFRT for palliative treatment were excluded from this analysis.

Note:

Percentages are percent of the total number of 28 responses. Responders could choose more than one answer option.

^1)^ One other response indicated individualized dosing per the specific case (no details given).

**Qu 8: Do you combine SFRT with conventionally fractionated external beam radiation (EBRT) for CURATIVE-intent treatment?  Check all that apply**

| **Answer choices** | **Responses**  Percentage No. | |
| --- | --- | --- |
| No, I treat with SFRT alone | 9.5% | 2 |
| Yes, SFRT before conventional EBRT | 57.1% | 12 |
| Yes, SFRT concurrent (interdigitated) with conventional EBRT | 19.0% | 4 |
| Yes, SFRT after conventional EBRT | 9.5% | 2 |
| Other (Specify) ^1)^ | 4.8% | 1 |
| Total | 99.9% | 21 |

Check box question. Answered by 25 radiation oncologists; 4 responders, who indicated they did not use SFRT for curative treatment were excluded from this analysis.

Note:

Percentages are percent of the total number of 21 responses. Responders could choose more than one answer option.

^1)^ One “Other” response did not specify a sequence.

**Qu 9: If you combine SFRT with conventionally fractionated external beam radiation for CURATIVE-intent, do you reduce the conventionally fractionated external beam radiation dose (compared to the dose for patients treated without SFRT)?**

| **Answer choices** | **Radiation Oncologists**  Percentage No. | |
| --- | --- | --- |
| No | 81.0% | 17 |
| Yes:  (Specify, estimate percentage by how much the conventionally fractionated external beam radiation dose is reduced) ^1)^ | 19.0% | 4 |
| Total | 100% | 21 |

Multiple choice question. Answered by 22 radiation oncologists. One responder, who indicated not to use SFRT for curative treatment was excluded from this analysis.

Note:

^1)^ The conventionally fractionated external beam radiation dose was reduced by 20% (2 responses), 20-50% (1 response), and reduced to 50 Gy/25 fractions (1 response).

**D. SFRT combination with systemic therapy**

(Questions to all radiation oncologists)

**Qu 10: Do you combine SFRT with chemotherapy for PALLIATVE treatment?**

| **Answer choices** | **Radiation Oncologists**  Percentage No. | |
| --- | --- | --- |
| No, chemotherapy only before the start and after completion of the radiation therapy course | 45.8% | 11 |
| Yes, chemotherapy only during the conventionally fractionated portion of treatment | 37.5% | 9 |
| Yes, chemotherapy during both the SFRT and the conventionally fractionated portion of treatment | 12.5% | 3 |
| Other (Specify) ^1)^ | 4.2% | 1 |
| Total | 100% | 24 |

Multiple choice question. Answered by 24 radiation oncologists.

Note:

^1)^ One responder individualized based on the primary tumor and general status of the patient.

**Qu 11: Do you combine SFRT with chemotherapy for CURATIVE-intent treatment?**

| **Answer choices** | **Radiation Oncologists**  Percentage No. | |
| --- | --- | --- |
| No, chemotherapy only before the start and after completion of the radiation therapy course | 45.5% | 10 |
| Yes, chemotherapy only during the conventionally fractionated portion of treatment | 45.5% | 10 |
| Yes, chemotherapy during both the SFRT and the conventionally fractionated portion of treatment | 9.1% | 2 |
| Other (Specify) | 0% | 0 |
| Total | 100% | 22 |

Multiple choice question. Answered by 22 radiation oncologists.

**Qu 12: Do you combine SFRT with immunotherapy for PALLIATIVE treatment?**

| **Answer choices** | **Radiation Oncologists**  Percentage No. | |
| --- | --- | --- |
| No, immunotherapy only before the start and after completion of the radiation therapy course | 55.0% | 11 |
| Yes, immunotherapy only during the conventionally fractionated portion of treatment | 10.0% | 2 |
| Yes, immunotherapy during both the SFRT and the conventionally fractionated portion of treatment | 25.0% | 5 |
| Other (Specify) ^1)^ | 10.0% | 2 |
| Total | 100% | 20 |

Multiple choice question. Answered by 21 radiation oncologists. One responder, who indicated not to use SFRT for palliative treatment was excluded from this analysis.

Note:

^1)^ One responder indicated no experience with immunotherapy in the responder’s practice to date; one gave no comment.

**Qu 13: Do you combine SFRT with immunotherapy for CURATIVE-intent treatment?**

| **Answer choices** | **Radiation Oncologists**  Percentage No. | |
| --- | --- | --- |
| No, immunotherapy only before the start and after completion of the radiation therapy course | 52.5% | 11 |
| Yes, immunotherapy only during the conventionally fractionated portion of treatment | 9.5% | 2 |
| Yes, immunotherapy during both the SFRT and the conventionally fractionated portion of treatment | 19.0% | 4 |
| Other (Specify) ^1)^ | 19.0% | 4 |
| Total | 100.0% | 21 |

Multiple choice question. Answered by 21 radiation oncologists.

Note:

^1)^ Three responders indicated no experience with immunotherapy in their practice to date; one gave no comment.

**E. SFRT Technology and technique**

(Questions to all radiation oncologists)

**Qu 14: What technique do you use for SFRT?  Check all that apply**

| **Answer choices** | **Responses**  Percentage No. | |
| --- | --- | --- |
| GRID therapy (collimator-based) | 22.5% | 9 |
| GRID therapy (MLC-based) | 27.5% | 11 |
| Lattice therapy | 45.0% | 18 |
| Other technique (Specify) ^1)^ | 5.0% | 2 |
| Total | 100% | 40 |

Check box question. Answered by 21 radiation oncologists.

Note:

Percentages are percent of the total number of 40 responses. Responders could choose more than one answer option. Four of 21 responders used both, GRID and Lattice therapy technique, and all of these used MLC-based GRID therapy.

^1)^ One response indicated proton beam GRID, and one immune-sparing radiotherapy (modified SBRT PATHY).

**Qu 15: Which equipment/technology do you use for SFRT?  Check all that apply**

| **Answer choices** | **Responses**  Percentage No. | |
| --- | --- | --- |
| Linear accelerator using GRID block | 16.1% | 9 |
| Linear accelerator using multi-leaf collimator for  GRID therapy | 17.9% | 10 |
| Linear accelerator using electronic compensation  for GRID therapy | 0% | 0 |
| Linear accelerator using VMAT for Lattice therapy | 25.0% | 14 |
| Linear accelerator using IMRT for Lattice therapy | 17.9% | 10 |
| Linear accelerator using electronic compensation  for Lattice therapy | 0% | 0 |
| Tomotherapy for Lattice therapy | 5.4% | 3 |
| Cyberknife for Lattice therapy | 8.9% | 5 |
| Proton therapy for GRID therapy | 5.4% | 3 |
| Carbon therapy for GRID or Lattice | 0% | 0 |
| Other (Specify) ^1)^ | 3.6% | 2 |
| Total | 100.2% | 56 |

Check box question. Answered by 27 radiation oncologists.

Note:

Percentages are percent of the total number of 56 responses. Responders could choose more than one answer option.

^1)^ Two responses indicated linear accelerators and did not specify the answer choice for SFRT technique.

**F. Regulatory considerations, research**

(Questions to all radiation oncologists)

**Qu 23: Are your patients treated with SFRT on an IRB (ethics committee) approved research protocol or do you use SFRT as a standard of care?**

| **Answer choices** | **Radiation Oncologists**  Percentage No. | |
| --- | --- | --- |
| on an IRB-approved protocol | 22.7% | 5 |
| as standard of care treatment | 72.7% | 16 |
| Both: Please specify which patients or diseases are treated on an IRB approved protocol and which are treated as  standard of care: | 4.5% | 1 |
| Total | 100% | 22 |

Multiple choice question. Answered by 22 radiation oncologists.

**Qu 24: Do you currently enrol patients into formal registered clinical trial(s)?**

| **Answer choices** | **Radiation Oncologists**  Percentage No. | |
| --- | --- | --- |
| No | 45.8% | 11 |
| Yes | 37.5% | 9 |
| I plan to in the next 1-2 years | 16.7% | 4 |
| Total |  | 24 |

Multiple choice question. Answered by 24 radiation oncologists.

**Qu 25: Have you published your clinical outcome data on SFRT or presented them at a scientific meeting?**

| **Answer choices** | **Radiation oncologists**  Percentage No. | |
| --- | --- | --- |
| No | 60.0% | 15 |
| Yes | 40.0% | 10 |
| Total | 100.0% | 25 |

Multiple choice question. Answered by 25 radiation oncologists.

**Qu 26: Do you have access to a radiobiologist or biologist in your center/institution or in a collaborating institution?**

| **Answer choices** | **Radiation oncologists**  Percentage No. | |
| --- | --- | --- |
| No | 40.0% | 10 |
| Yes | 52.0% | 13 |
| Other (Specify) ^1)^ | 8.00% | 2 |
| Total |  | 25 |

Multiple choice question. Answered by 25 radiation oncologists.

Note:

^1)^ Two responders indicate their centers anticipate having a radiobiologist or biologist.

**G. GRID therapy – treatment planning**

(Question to GRID therapy practitioners)

**Qu 16: When using GRID therapy, where do you prescribe the dose for GRID therapy planning?**

| **Answer choices** | **Radiation Oncologists (using GRID)**  Percentage No. | | |
| --- | --- | --- | --- |
| at Dmax | | 37.5% | 6 |
| at tumor depth | | 0% | 0 |
| to gross tumor volume (GTV) with an additional margin | | 0% | 0 |
| to gross tumor volume (GTV) without additional margin | | 62.5% | 10 |
| Other (specify) | | 0% | 0 |
| Total | | 100.0% | 16 |

Check box question. Answered by 16 radiation oncologists, who were GRID therapy users.

Note:

Percentages are percent of the total number of 16 responses. Responders could choose more than one answer option (account for variable dose prescription methods by the same responder for different clinical situations). For this question each responder gave only one answer.

**H. Lattice therapy – treatment planning**

(Question to Lattice therapy practitioners)

**Qu 19: When using LATTICE therapy, where do you prescribe the dose for Lattice therapy planning?**

| **Answer choices** | **Radiation Oncologists**  Percentage No. | |
| --- | --- | --- |
| at Dmax | 16.7% | 3 |
| to the volume of the vertices  (vertex tumor volume) | 44.4% | 8 |
| to gross tumor volume (GTV)  with an additional margin | 5.6% | 1 |
| to gross tumor volume (GTV) without additional margin | 27.8% | 5 |
| Other (Specify) ^1)^ | 5.6% | 1 |
| Total | 100.1% | 18 |

Check box question. Answered by 14 radiation oncologists, who were Lattice therapy users.

Note:

Percentages are percent of the total number of 18 responses. Responders could choose more than one answer option (to account for variable dose prescription methods by the same responder for different clinical situations.

^1)^ One responder indicated prescribing to the GTV with an inside margin (GTV minus an inner margin).

**I.** **Use of dosimetric parameters and attributed influence of dosimetric parameters on local control**

(Questions to GRID therapy practitioners and Lattice therapy practitioners)

**Qu 17: When using GRID therapy, how commonly do you use the following dosimetric parameters for GRID therapy planning?**

| **Parameter** | **Always** | **Often** | **Some- times** | **Rarely** | **Not at all** | **Did not respond *** | **Total** | **Weighted Average** |
| --- | --- | --- | --- | --- | --- | --- | --- | --- |
| GRID pre- scription dose | 93.3% 14 | 0.0% 0 | 0.0% 0 | 0.0% 0 | 0.0% 0 | 6.7% 1 | 15 | 1.3 |
| Dose volume histogram (DVH) | 66.7% 10 | 6.7% 1 | 13.3% 2 | 6.7% 1 | 6.7% 1 | 0.0% 0 | 15 | 1.8 |
| Dose to GTV margin | 20.0% 3 | 0.0% 0 | 13.3% 2 | 20.0% 3 | 33.3% 5 | 13.3% 2 | 15 | 3.9 |
| Valley dose (V) | 33.3% 5 | 13.3% 2 | 13.3% 2 | 0.0% 0 | 26.7% 4 | 13.3% 2 | 15 | 3.1 |
| Peak dose (P) | 46.7% 7 | 26.7% 4 | 6.7% 1 | 0.0% 0 | 13.3% 2 | 6.7% 1 | 15 | 2.3 |
| Valley/peak dose ratio (VPDR) or Peak/valley dose ratio (PVDR) | 26.7% 4 | 13.3% 2 | 13.3% 2 | 6.7% 1 | 26.7% 4 | 13.3% 2 | 15 | 3.3 |
| Dose covering 5% or 10% of PTV (D5 or D10) | 6.7% 1 | 13.3% 2 | 13.3% 2 | 6.7% 1 | 46.7% 7 | 13.3% 2 | 15 | 4.1 |
| Dose covering 50% of PTV (D50) | 6.7% 1 | 6.7% 1 | 6.7% 1 | 13.3% 2 | 53.3% 8 | 13.3% 2 | 15 | 4.4 |
| Dose covering 90% or 95% of PTV (D90 or D95) | 33.3% 5 | 0.0% 0 | 6.7% 1 | 6.7% 1 | 46.7% 7 | 6.7% 1 | 15 | 3.5 |
| Mean dose | 26.7% 4 | 6.7% 1 | 13.3% 2 | 6.7% 1 | 33.3% 5 | 13.3% 2 | 15 | 3.5 |
| Volume receiving 5 Gy or receiving 8 Gy per fraction | 20.0% 3 | 6.7% 1 | 13.3% 2 | 6.7% 1 | 46.7% 7 | 6.7% 1 | 15 | 3.7 |
| Peak-to-peak distance | 20.0% 3 | 20.0% 3 | 13.3% 2 | 6.7% 1 | 33.3% 5 | 6.7% 1 | 15 | 3.3 |
| Peak width (defined at 50% of the maximal dose) | 6.7% 1 | 6.7% 1 | 13.3% 2 | 13.3% 2 | 46.7% 7 | 13.3% 2 | 15 | 4.3 |
| Equivalent uniform dose (EUD) of the SFRT, computed with the LQ Model | 26.7% 4 | 6.7% 1 | 13.3% 2 | 6.7% 1 | 40.0% 6 | 6.7% 1 | 15 | 3.5 |
| Equivalent uniform dose (EUD) of the SFRT, computed with the generalized LQ Model | 26.7% 4 | 6.7% 1 | 6.7% 1 | 6.7% 1 | 46.7% 7 | 6.7% 1 | 15 | 3.6 |

*Practitioners who responded to at least one item were included in the calculations.

This question contains the recommended dosimetric parameters based on recent consensus recommendations for GRID dosimetric parameters use ^18^. The dosimetric parameters were classified in the categories presented in the table “Dosimetric parameters: Comparison of GRID and Lattice practitioners” (following question 21), as *classic*, *heterogeneity*, *geometric* and *biologic* parameters.

Matrix/rating scale question. The weighted average column was calculated after converting the categories to numbers from 1 (always) to 6 (did not respond), where the average was weighted by the number of responses with that category. The question was answered by 15 of the radiation oncologists, who identified as GRID therapy users.

The graphic display of the results and the comparison of dosimetric parameter use between GRID therapy and Lattice therapy are presented in Figure 3a of the manuscript.

**Qu 18: In patients you treat with GRID therapy, have you observed that any of the following parameters may influence local tumor control?**

| **Parameter** | **Yes** | **No** | **Not sure** | **Not using this parameter** | **Did not respond*** | **Total** | **Weighted Average** |
| --- | --- | --- | --- | --- | --- | --- | --- |
| GRID prescription dose | 64.3% 9 | 7.1% 1 | 28.6% 4 | 0.0% 0 | 0.0% 0 | 14 | 1.6 |
| Number of SFRT fractions (1 vs more than 1) | 21.4% 3 | 14.3% 2 | 21.4% 3 | 35.7% 5 | 7.1% 1 | 14 | 2.9 |
| Dose to GTV margin | 21.4% 3 | 0.0% 0 | 42.9% 6 | 28.6% 4 | 7.1% 1 | 14 | 3.0 |
| Valley dose (V) | 14.3% 2 | 14.3% 2 | 28.6% 4 | 28.6% 4 | 14.3% 2 | 14 | 3.1 |
| Peak dose (P) | 14.3% 2 | 14.3% 2 | 28.6% 4 | 28.6% 4 | 14.3% 2 | 14 | 3.1 |
| Valley/peak dose ratio (VPDR) or Peak/valley dose ratio (PVDR) | 14.3% 2 | 7.1% 1 | 35.7% 5 | 28.6% 4 | 14.3% 2 | 14 | 3.2 |
| Dose covering 5% or 10% of PTV (D5 or D10) | 7.1% 1 | 7.1% 1 | 35.7% 5 | 35.7% 5 | 14.3% 2 | 14 | 3.4 |
| Dose covering 50% of PTV (D50) | 7.1% 1 | 0.0% 0 | 35.7% 5 | 42.9% 6 | 14.3% 2 | 14 | 3.6 |
| Dose covering 90% or 95% of PTV (D90 or D95) | 21.4% 3 | 0.0% 0 | 28.6% 4 | 42.9% 6 | 7.1% 1 | 14 | 3.1 |
| Mean dose | 28.6% 4 | 0.0% 0 | 28.6% 4 | 28.6% 4 | 14.3% 2 | 14 | 3.0 |
| Volume receiving 5 Gy or receiving 8 Gy per fraction | 14.3% 2 | 7.1% 1 | 21.4% 3 | 42.9% 6 | 14.3% 2 | 14 | 3.4 |
| Peak-to-peak distance | 14.3% 2 | 7.1% 1 | 28.6% 4 | 35.7% 5 | 14.3% 2 | 14 | 3.3 |
| Peak width (defined at 50% of the maximal dose) | 7.1% 1 | 7.1% 1 | 35.7% 5 | 35.7% 5 | 14.3% 2 | 14 | 3.4 |
| Conventional external beam radiation dose (given in addition to SFRT) | 71.4% 10 | 0.0% 0 | 21.4% 3 | 0.0% 0 | 7.1% 1 | 14 | 1.7 |
| Equivalent uniform dose (EUD) of the SFRT | 42.9% 6 | 0.0% 0 | 21.4% 3 | 28.6% 4 | 7.1% 1 | 14 | 2.6 |
| Timing of SFRT dose (before, during or after) conventional radiation (please specify below) | 35.7% 5 | 7.1% 1 | 42.9% 6 | 7.1% 1 | 7.1% 1 | 14 | 2.4 |

*Practitioners who responded to at least one item were included in the calculations.

Matrix/rating scale question. The weighted average column was calculated after converting the categories to numbers from 1 (always) to 6 (did not respond), where the average was weighted by the number of responses with that category. The question was answered by 14 of the radiation oncologists, who identified as GRID therapy users.

The question contained three additional treatment planning parameters (beyond those of the previous question), that were examined for their influence on outcome attributed by the practitioners. These *treatment strategy* parameters (see Table “Dosimetric parameters: Comparison of GRID and Lattice practitioners” following question 21) included number of SFRT fractions (1 vs. >1), timing of SFRT dose (before, during or after) conventional radiation, and conventional external beam radiation dose (given in addition to SFRT).

The graphic display of the results and the comparison between GRID therapy and Lattice therapy are presented in Figure 3b of the manuscript.

**Qu 20: When using LATTICE therapy, how commonly do you use the following dosimetric parameters for Lattice therapy planning?**

| **Parameter** | **Always** | **Often** | **Some- times** | **Rarely** | **Not at all** | **Did not respond *** | **Total** | **Weighted Average** |
| --- | --- | --- | --- | --- | --- | --- | --- | --- |
| Lattice prescription dose | 93.3% 14 | 0.0% 0 | 0.0% 0 | 6.7% 1 | 0.0% 0 | 0.0% 0 | 15 | 1.2 |
| Dose volume histogram (DVH) | 80.0% 12 | 0.0% 0 | 13.3% 2 | 0.0% 0 | 6.7% 1 | 0.0% 0 | 15 | 1.5 |
| Dose to GTV margin | 46.7% 7 | 6.7% 1 | 0.0% 0 | 6.7% 1 | 13.3% 2 | 26.7% 4 | 15 | 3.1 |
| Valley dose | 66.7% 10 | 0.0% 0 | 6.7% 1 | 0.0% 0 | 13.3% 2 | 13.3% 2 | 15 | 2.3 |
| Peak dose | 60.0% 9 | 6.7% 1 | 6.7% 1 | 0.0% 0 | 13.3% 2 | 13.3% 2 | 15 | 2.4 |
| D5/D95 ratio or D10/D90 ratio | 53.3% 8 | 0.0% 0 | 0.0% 0 | 0.0% 0 | 26.7% 4 | 20.0% 3 | 15 | 3.1 |
| Mean dose | 33.3% 5 | 13.3% 2 | 6.7% 1 | 6.7% 1 | 20.0% 3 | 20.0% 3 | 15 | 3.3 |
| Volume receiving 5 Gy or receiving 8 Gy per fraction | 60.0% 9 | 6.7% 1 | 0.0% 0 | 6.7% 1 | 20.0% 3 | 6.7% 1 | 15 | 2.4 |
| Volume to vertices / Volume to GTV (volume ratio) | 53.3% 8 | 13.3% 2 | 0.0% 0 | 13.3% 2 | 13.3% 2 | 6.7% 1 | 15 | 2.4 |
| Vertices diameter | 66.7% 10 | 13.3% 2 | 0.0% 0 | 6.7% 1 | 13.3% 2 | 0.0% 0 | 15 | 1.9 |
| Vertices separation | 66.7% 10 | 13.3% 2 | 6.7% 1 | 6.7% 1 | 6.7% 1 | 0.0% 0 | 15 | 1.7 |
| Number of vertices | 66.7% 10 | 13.3% 2 | 0.0% 0 | 13.3% 2 | 6.7% 1 | 0.0% 0 | 15 | 1.8 |
| Equivalent uniform dose (EUD) of the SFRT, computed with the LQ Model | 26.7% 4 | 0.0% 0 | 6.7% 1 | 13.3% 2 | 26.7% 4 | 26.7% 4 | 15 | 3.9 |
| Equivalent uniform dose (EUD) of the SFRT, computed with the generalized LQ Model | 33.3% 5 | 0.0% 0 | 0.0% 0 | 20.0% 3 | 20.0% 3 | 26.7% 4 | 15 | 3.7 |

*Practitioners who responded to at least one item were included in the calculations.

Matrix/rating scale question. The weighted average column was calculated after converting the categories to numbers from 1 (always) to 6 (did not respond), where the average was weighted by the number of responses with that category. The question was answered by 15 of the radiation oncologists who were Lattice therapy users.

This question contains the recommended dosimetric parameters based on recent consensus recommendations for Lattice therapy dosimetric parameters use ^17^. The dosimetric parameters were classified into the categories presented in the table “Dosimetric parameters: Comparison of GRID and Lattice practitioners” (following question 21) as *classic*, *heterogeneity*, *geometric* and *biologic* parameters.

The graphic display of the results and the comparison of dosimetric parameter use between Lattice therapy and GRID therapy are presented in Figure 3a of the manuscript.

**Qu 21: In patients you treat with LATTICE therapy, have you observed that any of the following parameters may influence local tumor control?**

| **Parameter** | **Yes** | **No** | **Not sure** | **Not using this parameter** | **Did not respond*** | **Total** | **Weighted Average** |
| --- | --- | --- | --- | --- | --- | --- | --- |
| Lattice pre-scription dose | 73.3% 11 | 6.7% 1 | 20.0% 3 | 0.0% 0 | 0.0% 0 | 15 | 1.5 |
| Number of SFRT fractions (1 vs more  than 1) | 53.3% 8 | 13.3% 2 | 20.0% 3 | 6.7% 1 | 6.7% 1 | 15 | 2.0 |
| Dose to GTV margin | 26.7% 4 | 40.0% 6 | 13.3% 2 | 6.7% 1 | 13.3% 2 | 15 | 2.4 |
| Valley dose | 46.7% 7 | 13.3% 2 | 20.0% 3 | 6.7% 1 | 13.3% 2 | 15 | 2.3 |
| Peak dose | 53.3% 8 | 6.7% 1 | 20.0% 3 | 6.7% 1 | 13.3% 2 | 15 | 2.2 |
| D5/D95 ratio or D10/D90 ratio | 33.3% 5 | 26.7% 4 | 13.3% 2 | 6.7% 1 | 20.0% 3 | 15 | 2.5 |
| Mean dose | 26.7% 4 | 33.3% 5 | 13.3% 2 | 6.7% 1 | 20.0% 3 | 15 | 2.6 |
| Volume receiving 5 Gy or receiving 8 Gy per fraction | 26.7% 4 | 20.0% 3 | 20.0% 3 | 6.7% 1 | 26.7% 4 | 15 | 2.9 |
| Volume to vertices / volume to GTV (volume ratio) | 46.7% 7 | 26.7% 4 | 26.7% 4 | 0.0% 0 | 0.0% 0 | 15 | 1.8 |
| Vertices diameter | 46.7% 7 | 20.0% 3 | 26.7% 4 | 0.0% 0 | 6.7% 1 | 15 | 2.0 |
| Vertices separation | 33.3% 5 | 26.7% 4 | 26.7% 4 | 0.0% 0 | 13.3% 2 | 15 | 2.3 |
| Number of vertices | 53.3% 8 | 13.3% 2 | 33.3% 5 | 0.0% 0 | 0.0% 0 | 15 | 1.8 |
| Conventional external beam radiation dose (given in addition to SFRT) | 60.0% 9 | 6.7% 1 | 20.0% 3 | 6.7% 1 | 6.7% 1 | 15 | 1.9 |
| Equivalent uniform dose (EUD) of the SFRT | 33.3% 5 | 20.0% 3 | 13.3% 2 | 20.0% 3 | 13.3% 2 | 15 | 2.6 |
| Timing of SFRT dose (before, during or after) conventional radiation(please specify below) | 33.3% 5 | 20.0% 3 | 13.3% 2 | 20.0% 3 | 13.3% 2 | 15 | 2.6 |

*Practitioners who responded to at least one item were included in the calculations.

As for GRID therapy, the question contained the three additional treatment planning parameters described for GRID therapy.

Matrix/rating scale question. The weighted average column was calculated after converting the categories to numbers from 1 (always) to 6 (did not respond), where the average was weighted by the number of responses with that category. The question was answered by 15 of the radiation oncologists who were Lattice therapy users.

This question contains the recommended dosimetric parameters based on recent consensus recommendations for GRID dosimetric parameters use ^18^. The dosimetric parameters were classified in the categories presented in the table “Dosimetric parameters: Comparison of GRID and Lattice practitioners” (below), as *classic*, *heterogeneity*, *geometric* and *biologic* parameters.

The graphic display of the results and the comparison between Lattice therapy and GRID therapy are presented in Figure 3b of the manuscript.

**Table:** **Dosimetric parameters: Comparison of GRID and Lattice practitioners:**

Comparison findings for questions 17 vs. 20 and questions 18 vs. 21

Classification of parameters

**
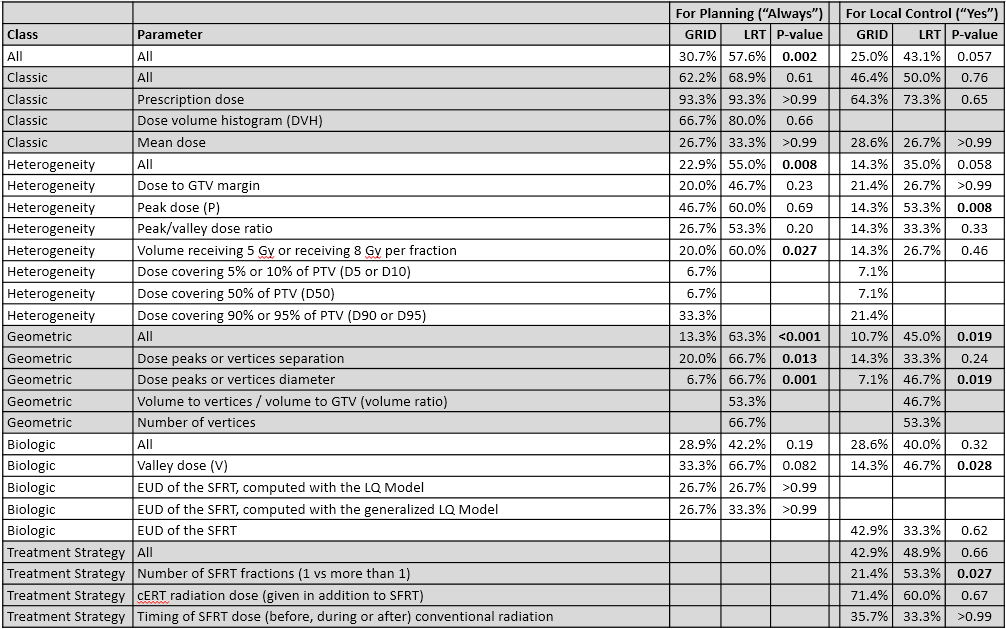
**

Note:

Parameters that are comparable between GRID and Lattice therapy include:

Classic parameters: GRID prescription dose, Dose volume histogram (DVH), and Mean dose;

Heterogeneity parameters: Peak dose (P), Valley/peak dose ratio (VPDR) or Peak/valley dose ratio (PVDR), Dose to GTV margin, and Volume receiving 5 Gy or receiving 8 Gy per fraction;

Geometric parameters: Peak width (defined at 50% of the maximal dose), Peak-to-peak distance; and

Biologic parameters: Valley dose (V), Equivalent uniform dose (EUD) of the SFRT, computed with the LQ Model, and Equivalent uniform dose (EUD) of the SFRT, computed with the generalized LQ Mode.

The remainder of the parameters are not comparable between GRID and Lattice therapy due to differences in current technique and standards of parameter reporting among the two SFRT techniques (no data for these parameters in the table).

**J. Dosimetric parameters: Valley dose**

(Question to all radiation oncologists)

**Qu 22: What do you believe is an appropriate Valley Dose (i.e. low-dose within the tumor target)?**

| **Answer choices** | **Radiation Oncologists**  Percentage No. | |
| --- | --- | --- |
| 5 Gy in 1 fraction | 20.8% | 5 |
| 8 Gy in 1 fraction | 4.2% | 1 |
| as low as achievable | 58.3% | 14 |
| Valley dose is not important | 0% | 0 |
| Unsure | 16.7% | 4 |
| Other (Specify): | 0% | 0 |
| Total | 100% | 24 |

Multiple choice question. Answered by 24 radiation oncologists.

**K. Physicists’ use of technology, treatment planning, dosimetric parameters**

**and QA**

(Questions to physicists)

**Qu 27: Which equipment/technology do you use for SFRT?  Check all that apply**

| **Answer choices** | **Responses**  Percentage No. | |
| --- | --- | --- |
| Linear accelerator using GRID block | 18.2% | 4 |
| Linear accelerator using multi-leaf collimator for GRID therapy | 18.2% | 4 |
| Linear accelerator using electronic compensation for GRID therapy | 4.5% | 1 |
| Linear accelerator using VMAT for Lattice therapy | 27.3% | 6 |
| Linear accelerator using IMRT for Lattice therapy | 9.1% | 2 |
| Linear accelerator using electronic compensation for Lattice therapy | 4.5% | 1 |
| Tomotherapy for Lattice therapy | 0% | 0 |
| Cyberknife for Lattice therapy | 4.5% | 1 |
| Proton therapy for GRID therapy | 13.6% | 3 |
| Carbon therapy for GRID or Lattice | 0% | 0 |
| Other (Specify) | 0% | 0 |
| Total | 99.9% | 22 |

Check box question. Answered by 9 physicists.

Note:

Percentages are percent of the total number of 22 responses. Responders could choose more than one answer option.

**Qu 28: Have you performed commissioning for a GRID block or collimator (including beam profile scanning at Dmax, output measurement at different depths for different field sizes, peak-valley dose ratio measurement at different depths)?**

| **Answer choices** | **Physicists**  Percentage No. | |
| --- | --- | --- |
| No | 33.3% | 3 |
| Yes | 66.7% | 6 |
| I plan to in the next 1-2 years | 0% | 0 |
| Total | 100% | 9 |

Multiple choice question. Answered by 9 physicists.

**Qu 29: Do you or physicist colleagues in your clinic/center perform patient-specific QA for SFRT?**

| **Answer choices** | **Physicists**  Percentage No. | |
| --- | --- | --- |
| No | 0% | 0 |
| Yes | 100% | 8 |
| I plan to in the next 1-2 years | 0% | 0 |
| Total | 100% | 8 |

Multiple choice question. Answered by 8 physicists.

**Qu 30: Which equipment do you use for patient-specific QA for SFRT?  Check all that apply**

| **Answer choices** | **Responses**  Percentage No. | |
| --- | --- | --- |
| Film | 26.3% | 5 |
| Ion chamber | 21.1% | 4 |
| MapCheck | 5.3% | 1 |
| ArcCheck | 15.8% | 3 |
| Portal dosimetry | 21.1% | 4 |
| Other (Specify) ^1)^ | 10.5% | 2 |
| Total | 100.1% | 19 |

Check box question. Answered by 9 physicists.

Note:

Percentages are percent of the total number of 19 responses. Responders could choose more than one answer option.

^1)^ One responder indicated MatriXX-PT, and one ScandiDos Delta4 and PTW Octavius + SRS1000.

**Qu 31: Which planning system(s) do you use to generate SFRT plans?   Check all that apply**

| **Answer choices** | **Responses**  Percentage No. | |
| --- | --- | --- |
| Eclipse | 46.7% | 7 |
| Pinnacle | 13.3% | 2 |
| RaySearch Station | 20.0% | 3 |
| Own developed planning software | 0% | 0 |
| Other (Specify) ^1)^ | 20.0% | 3 |
| Total | 100% | 15 |

Check box question. Answered by 9 physicists.

Note:

Percentages are percent of the total number of 15 responses. Responders could choose more than one answer option.

^1)^ Two responses indicated Monaco and one Accuray Cyberknife MultiPlan as planning system.

**Qu 32: How commonly do you provide the following dosimetric parameters to the physician for GRID therapy planning? Check all that apply**

| **Parameter** | **Always** | **Often** | **Some-times** | **Rarely** | **Not at all** | **Did not respond*** | **Total** | **Weighted Average** |
| --- | --- | --- | --- | --- | --- | --- | --- | --- |
| Dose volume histogram (DVH) | 75.0% 6 | 0% 0 | 0% 0 | 0% 0 | 12.5% 1 | 12.5% 1 | 8 | 2.1 |
| Dose to GTV margin | 37.5% 3 | 12.5% 1 | 0% 0 | 12.5% 1 | 25.0% 2 | 12.5% 1 | 8 | 3.1 |
| Valley dose (V) | 37.5% 3 | 25.0% 2 | 0% 0 | 12.5% 1 | 25.0% 2 | 0% 0 | 8 | 2.6 |
| Peak dose (P) | 37.5% 3 | 25.0% 2 | 0% 0 | 12.5% 1 | 25.0% 2 | 0% 0 | 8 | 2.6 |
| Valley/peak dose ratio (VPDR) or Peak/valley dose ratio (PVDR) | 37.5% 3 | 0% 0 | 12.5% 1 | 25.0% 2 | 25.0% 2 | 0% 0 | 8 | 3.0 |
| Peak-to-peak distance | 37.5% 3 | 12.5% 1 | 0% 0 | 0% 0 | 25.0% 2 | 25.0% 2 | 8 | 3.4 |
| Peak width (defined at 50% of the maximal dose) | 25.0% 2 | 0% 0 | 12.5% 1 | 12.5% 1 | 25.0% 2 | 25.0% 2 | 8 | 3.9 |
| Dose covering 5% or 10% of PTV volumes (D5 or D10) | 25.0% 2 | 0% 0 | 12.5% 1 | 0% 0 | 50.0% 4 | 12.5% 1 | 8 | 3.9 |
| Dose covering 50% of PTV (D50) | 50.0% 4 | 12.5% 1 | 0% 0 | 0% 0 | 25.0% 2 | 12.5% 1 | 8 | 2.8 |
| Dose covering 90% or 95% of PTV volume (D90 or D95) | 37.5% 3 | 0% 0 | 12.5% 1 | 0% 0 | 37.5% 3 | 12.5% 1 | 8 | 3.4 |
| Mean dose | 50.0% 4 | 12.5% 1 | 25.0% 2 | 0% 0 | 12.5% 1 | 0% 0 | 8 | 2.1 |
| Volume receiving 5 Gy and/or 8 Gy per fraction | 25.0% 2 | 0% 0 | 25.0% 2 | 12.5% 1 | 25.0% 2 | 12.5% 1 | 8 | 3.5 |
| Equivalent uniform dose (EUD) of the SFRT, computed with the LQ Model | 0% 0 | 12.5% 1 | 12.5% 1 | 12.5% 1 | 50.0% 4 | 12.5% 1 | 8 | 4.4 |
| Equivalent uniform dose (EUD) of the SFRT, computed with the generalized LQ Model | 12.5% 1 | 12.5% 1 | 12.5% 1 | 12.5% 1 | 50.0% 4 | 0% 0 | 8 | 3.8 |

Matrix/rating scale question. The weighted average column was calculated after converting the categories to numbers from 1 (always) to 6 (did not respond), where the average was weighted by the number of responses with that category. Answered by 8 physicists.

This question contains the recommended dosimetric parameters based on recent consensus recommendations for GRID dosimetric parameters use ^18^ (see section I, question 17).

*Physicists who responded to at least one item were included in the calculations.

**Qu 33: In LATTICE therapy, do you pre-define the vertex locations and sizes?**

| **Answer choices** | **Physicists**  Percentage No. | |
| --- | --- | --- |
| No | 55.6% | 5 |
| Yes | 44.4% | 4 |
| Other (please specify): | 0% | 0 |
| Total | 100% | 9 |

Multiple choice question. Answered by 9 physicists.

**Qu 34: How commonly do you provide the following dosimetric parameters for LATTICE therapy planning? Check all that apply**

| **Parameter** | **Always** | **Often** | **Some-times** | **Rarely** | **Not at all** | **Did not respond*** | **Total** | **Weighted Average** |
| --- | --- | --- | --- | --- | --- | --- | --- | --- |
| Dose volume histogram (DVH) | 100% 8 | 0% 0 | 0% 0 | 0% 0 | 0% 0 | 0% 0 | 8 | 1.0 |
| Dose to GTV margin | 50.0% 4 | 12.5% 1 | 0% 0 | 12.5% 1 | 12.5% 1 | 12.5% 1 | 8 | 2.6 |
| Valley dose | 62.5% 5 | 25.0% 2 | 0% 0 | 12.5% 1 | 0% 0 | 0% 0 | 8 | 1.6 |
| Peak dose | 62.5% 5 | 25.0% 2 | 0% 0 | 12.5% 1 | 0% 0 | 0% 0 | 8 | 1.6 |
| D5/D95 or D10/D90 ratio | 25.0% 2 | 12.5% 1 | 12.5% 1 | 12.5% 1 | 37.5% 3 | 0% 0 | 8 | 3.2 |
| Mean dose | 50.0% 4 | 25.0% 2 | 12.5% 1 | 0% 0 | 12.5% 1 | 0% 0 | 8 | 2.0 |
| Volume receiving 5 Gy or receiving 8 Gy per fraction | 37.5% 3 | 0% 0 | 12.5% 1 | 12.5% 1 | 25.0% 2 | 12.5% 1 | 8 | 3.2 |
| Volume to vertices / Volume to GTV (volume ratio) | 37.5% 3 | 0% 0 | 25.0% 2 | 0% 0 | 12.5% 1 | 25.0% 2 | 8 | 3.2 |
| Vertices diameter | 62.5% 5 | 0% 0 | 12.5% 1 | 0% 0 | 0% 0 | 25.0% 2 | 8 | 2.5 |
| Vertices separation | 50.0% 4 | 0% 0 | 25.0% 2 | 0% 0 | 0% 0 | 25.0% 2 | 8 | 2.8 |
| Number of vertices | 37.5% 3 | 25.0% 2 | 25.0% 2 | 0% 0 | 0% 0 | 12.5% 1 | 8 | 2.4 |
| Equivalent uniform dose (EUD) of the SFRT, computed with the LQ Model | 0% 0 | 0% 0 | 25.0% 2 | 12.5% 1 | 37.5% 3 | 25.0% 2 | 8 | 4.6 |
| Equivalent uniform dose (EUD) of the SFRT, computed with the generalized LQ Model | 25.0% 2 | 0% 0 | 12.5% 1 | 12.5% 1 | 37.5% 3 | 12.5% 1 | 8 | 3.8 |

Matrix/rating scale question. The weighted average column was calculated after converting the categories to numbers from 1 (always) to 6 (did not respond), where the average was weighted by the number of responses with that category. Answered by 8 physicists.

This question contains the recommended dosimetric parameters based on recent consensus recommendations for Lattice therapy dosimetric parameters use ^17^, see section I, question 20).

*Physicists who responded to at least one item were included in the calculations.

**Qu 35: What do you believe is an appropriate Valley Dose (i.e. low-dose within the tumor target)?**

| **Answer choices** | **Physicists**  Percentage No. | |
| --- | --- | --- |
| 5 Gy in 1 fraction | 0% | 0 |
| 8 Gy in 1 fraction | 11.1% | 1 |
| as low as achievable | 55.6% | 5 |
| Valley dose is not important | 0% | 0 |
| unsure | 22.2% | 2 |
| Other (please specify) ^1)^ | 11.1% | 1 |
| Total | 100.0% | 9 |

Multiple choice question. Answered by 9 physicists.

Note:

^1)^ One responder indicated 18 to 20 Gy for a fractionation of 66.7 Gy in 5 fractions to high-dose spheres.

**L. Primary disease-specific questions: Head and neck cancer**

(Questions to radiation oncologists who practice SFRT for head and neck cancer)

Questions in this section were tailored according to recommendations in the SFRT literature for head and neck (H&N) cancer and according to areas of uncertainty or controversy.

**Qu 37: For SFRT in HEAD AND NECK cancer, what dose do you prescribe for the SFRT? Check all dose regimens you use**

**Qu 38: Please check all where your answer is "Yes": For SFRT in HEAD AND NECK cancer, do you treat the primary tumor with SFRT , …** (see Table 1 in manuscript for specific treatment criteria).

Check box questions. These questions were targeted to radiation oncologist who identified as practicing SFRT for primary H&N cancer in question 4 (‘*What type of tumors do you treat with SFRT? Check all that apply*’).

The results are presented in Table 1 of the manuscript.

**Qu 39: How much time do you typically allow between the SFRT dose and the start of conventional radiation in HEAD AND NECK cancer?**

| **Answer choices** | **Radiation Oncologists**  Percentage No. | |
| --- | --- | --- |
| 1 day | 35.7% | 5 |
| 1-3 days | 14.3% | 2 |
| 2-3 days | 14.3% | 2 |
| >3 days | 14.3% | 2 |
| Another time interval (Specify) ^1)^ | 21.4% | 3 |
| Total | 100.0% | 14 |

Check box question. Answered by 14 radiation oncologists who treat primary H&N cancer with SFRT.

Note:

This question was targeted to radiation oncologist who identified as practicing SFRT for primary H&N cancer in question 4 (‘*What type of tumors do you treat with SFRT? Check all that appl*y’).

^1)^ One responder indicated an 8-10 week gap for increased immunologic response; one responder indicated an 8-10 week gap for tumor size reduction; and one indicated no gap.

**M. Primary disease-specific questions: Lung cancer**

(Questions to radiation oncologists who practice SFRT for lung cancer)

Questions in this section were tailored according to recommendations in the SFRT literature for lung cancer and according to areas of uncertainty or controversy.

**Qu 40: For SFRT in LUNG cancer, what dose do you prescribe for the SFRT? Check all dose regimens you use**

**Qu 41: Please check all where your answer is "Yes": For SFRT in LUNG cancer, do you** treat stage IIIC (T3-4, N3 M0) lung cancer with SFRT?, …(see Table 2 in manuscript for specific treatment criteria)

Check box questions. These questions were targeted to radiation oncologist who identified as practicing SFRT for primary lung cancer in question 4 (‘*What type of tumors do you treat with SFRT? Check all that apply*”).

The results are presented in Table 2 of the manuscript.

**Qu 42: How much time do you typically allow between the SFRT dose and the start of conventional radiation in LUNG cancer?**

| **Answer choices** | **Radiation Oncologists**  Percentage No. | |
| --- | --- | --- |
| 1 day | 25.0% | 2 |
| 1-3 days | 25.0% | 2 |
| 2-3 days | 0.0% | 0 |
| >3 days | 12.5% | 1 |
| Another time interval (Specify) ^1)^ | 37.5% | 3 |
| Total | 100.0% | 8 |

Check box question. Answered by 8 radiation oncologist who practice SFRT for primary lung cancer.

Note:

This questions was targeted to radiation oncologist who identified as practicing SFRT for primary lung cancer in question 4 (‘*What type of tumors do you treat with SFRT? Check all that apply*’).

^1)^ One responder indicated a 3-4 week gap; one no gap; and one indicated an interdigitated schedule.

**N. Primary disease-specific questions: Cervical cancer**

(Questions to radiation oncologists who practice SFRT for cervical cancer)

Questions in this section were tailored according to recommendations in the SFRT literature for cervical cancer and according to areas of uncertainty or controversy.

**Qu 43: For SFRT in CERVICAL cancer, what dose do you prescribe for the SFRT? Check all dose regimens you use**

**Qu 44: Please check all where your answer is "Yes": For SFRT in CERVICAL cancer, do you** treat patients with tumor size >5 cm ?… Answer options are presented in the left column of the table.

Check box questions. These questions were targeted to radiation oncologist who identified as practicing SFRT for primary cervical cancer in question 4 (‘*What type of tumors do you treat with SFRT? Check all that apply*’).

Results for both questions are presented in the table (next page).

The data are limited by small number of answers (5).

| **Selection or Planning Criterion** | **Used by percentage**  **of responders** |
| --- | --- |
| ***Patient selection:*** | |
| Treat patients with tumor size >5 cm | 20.0% |
| Treat patients with tumor size >6-7 cm | 80.0% |
| Treat patients with anatomical distortion expected to preclude brachytherapy | 40.0% |
| ***SFRT technology:*** |  |
| Use Lattice | 40.0% |
| Use GRID | 20.0% |
| ***Dose prescription and planning:*** | |
| Prescription dose ^1)^: 15 Gy / 1 fraction | 40.0% |
| 24 Gy / 3 fraction | 20.0% |
| 30 Gy / 3 fraction | 20.0% |
| Use the GTV of the cervical tumor as the SFRT target | 40.0% |
| Use the GTV of the cervical tumor plus a margin as the SFRT target | 20.0% |
| Also treat bulky lymph node involvement with SFRT | 60.0% |
| Reduce the GTV of the cervical tumor to exclude normal tissues | 60.0% |
| Reduce the prescription dose of the conventional external beam radiation because of the SFRT dose | 20.0% |
| Reduce the normal tissue dose limits (for conventional external radiation) because of the SFRT dose | 0.0% |
| Use adaptive therapy replanning for tumor volume changes | 60.0% |
| ***Combination with systemic therapies:*** | |
| Allow chemotherapy on the day(s) of SFRT | 20.0% |
| Combine immunotherapy with SFRT | 0.0% |
| ***Brachytherapy and surgery:*** | |
| Add brachytherapy after SFRT and conventional external radiation | 20.0% |
| Add an external beam boost after SFRT and conventional external radiation | 40.0% |
| Allow hysterectomy after SFRT and conventional external radiation | 40.0% |

Note:

^1)^ The three most common prescription doses are shown. Single-fraction doses of 15 Gy / 1 fraction or 18 Gy were not used.

**Qu 45: How much time do you typically allow between the SFRT dose and the start of conventional radiation in CERVICAL cancer ?**

| **Answer choices** | **Radiation Oncologists**  Percentage No. | |
| --- | --- | --- |
| 1 day | 50.0% | 2 |
| 1-3 days | 25.0% | 1 |
| 2-3 days | 0% | 0 |
| >3 days | 0% | 0 |
| Another time interval (Specify) ^1)^ | 25.0% | 1 |
| Total | 100.0% | 4 |

Check box question. Answered by 4 radiation oncologist who practice SFRT for primary cervical cancer.

Note:

This question was targeted to radiation oncologist who identified as practicing SFRT for primary cervical cancer in question 4 (‘*What type of tumors do you treat with SFRT? Check all that apply*’).

^1)^ One responder indicated a 3-4 week gap.

The data are limited by small number of answers (4).

**O. Disease-specific questions to radiation oncologists: Sarcoma**

(Questions to radiation oncologists who practice SFRT for sarcoma)

Questions in this section were tailored according to recommendations in the SFRT literature for sarcoma and according to areas of uncertainty or controversy.

**Qu 46: For SFRT in SARCOMA, what dose do you prescribe for the SFRT ? Check all dose regimens you use**

**Qu 47: Please check all where your answer is "Yes": For SFRT in SARCOMA, do you** treat soft tissue sarcoma of the trunk/chest ?… (see Table 3 in manuscript for specific treatment criteria).

Check box questions. These questions were targeted to radiation oncologist who identified as practicing SFRT for primary cervical cancer in question 4 “*What type of tumors do you treat with SFRT? Check all that apply*”.

The results are presented in Table 4 of the manuscript.

**Qu 48: How much time do you typically allow between the SFRT dose and the start of conventional radiation in SARCOMA?**

| **Answer choices** | **Radiation Oncologists**  Percentage No. | |
| --- | --- | --- |
| 1 day | 12.5% | 1 |
| 1-3 days | 37.5% | 3 |
| 2-3 days | 0.0% | 0 |
| >3 days | 12.5% | 1 |
| Another time interval (Specify) ^1)^ | 37.5% | 3 |
| Total | 100% | 8 |

Check box question. Answered by 8 radiation oncologists who practice SFRT for primary sarcoma.

Note:

These questions were targeted to radiation oncologist who identified as practicing SFRT for primary cervical cancer in question 4 “*What type of tumors do you treat with SFRT? Check all that apply*”.

^1)^ Two responders (25%) indicated no gap; one responder indicated 4-6 weeks.

**P.** **Demographic Question: Education and Practice**

(Questions to radiation oncologists and physicists)

**Qu 50: Have you received training in SFRT in your professional training (e.g. in residency)?**

| **Answer choices** | **Radiation Oncologists or Physicists**  Percentage No. | |
| --- | --- | --- |
| No | 75.0% | 27 |
| Yes, in GRID therapy | 16.7% | 6 |
| Yes, in Lattice therapy | 8.3% | 3 |
| Yes, in SFRT using Proton beam | 0% | 0 |
| Other (Specify) | 0% | 0 |
| Total | 100% | 36 |

Check box question. Answered by 36 radiation oncologists or physicists.

**Qu 51: Demographic Question:  How many years (after completing radiation oncology or radiation oncology physics training) have you practiced radiation oncology or radiation oncology physics?**

| **Answer choices** | **Radiation Oncologists or Physicists**  Percentage No. | |
| --- | --- | --- |
| 0 - 9 | 20.6% | 7 |
| 10 - 19 | 50.0% | 17 |
| 20 - 29 | 17.6% | 6 |
| 30 or more | 11.8% | 4 |
| Total | 100% | 34 |

Multiple choice question. Answered by 34 radiation oncologists or physicists.

**Qu 52: Demographic Question:  How many years have you practiced SFRT ?**

| **Answer choices** | **Radiation Oncologists or Physicists**  Percentage No. | |
| --- | --- | --- |
| 0 - 9 | 72.7% | 24 |
| 10 - 19 | 21.2% | 7 |
| 20 - 29 | 6.1% | 2 |
| 30 or more | 0% | 0 |
| Total | 100% | 33 |

Multiple choice question. Answered by 33 radiation oncologists or physicists.

**Qu 53: Demographic Question:  Where do you practice?**

| **Answer choices** | **Radiation Oncologists or Physicists**  Percentage No. | |
| --- | --- | --- |
| Eastern United States | 20.6% | 7 |
| Mid-western United States | 20.6% | 7 |
| Southern United States | 17.7% | 6 |
| Western United States | 2.9% | 1 |
| U.S. territories and islands | 0% | 0 |
| International:  Please specify your country ^1)^ | 38.2% | 13 |
| Total | 100% | 34 |

Multiple choice question. Answered by 34 radiation oncologists or physicists.

Note:

^1)^ The distribution of the 13 international practitioners is illustrated as shown:

| **International SFRT Practitioners** | **Radiation Oncologists or Physicists**  Percentage No. | |
| --- | --- | --- |
| Asia | 38.5% | 5 |
| Europe | 30.8% | 4 |
| Latin America | 30.8% | 4 |
| Total international practitioners | 100.1% | 13 |

**Qu 54: Demographic Question:  What is your current type of practice?**

| **Answer choices** | **Radiation Oncologists or Physicists**  Percentage No. | |
| --- | --- | --- |
| Private practice | 17.6% | 6 |
| Academic hospital or university | 55.9% | 19 |
| Hybrid (academic and private practice) | 8.8% | 3 |
| Hospital employment | 11.8% | 4 |
| Government/military facility | 5.9% | 2 |
| Other | 0% | 0 |
| Total | 100% | 34 |

Multiple choice question. Answered by 34 radiation oncologists or physicists.

**Q. Questions to non-practitioners**

These questions were targeted to those who indicated that they did not practice SFRT or were radiobiologists, molecular biologists or related scientists.

**Qu 55: Do you believe that there is a biological rationale for the use of SFRT (GRID or Lattice therapy) in clinical patients to improve response to radiation?**

| **Answer choices** | **Nonpractitioners**  Percentage No. | |
| --- | --- | --- |
| Yes | 64.3% | 18 |
| No | 7.1 | 2 |
| Not sure | 28.6% | 8 |
| Total | 100% | 28 |

Multiple choice question. Answered by 34 non-practitioners.

Note:

Non-practitioners include radiation oncologists and physicists who do not practice SFRT, and radiobiologists, molecular biologists or related scientists.

**Qu 56: Do you have clinician colleague(s) in your institution or clinic who treat patients with SFRT?**

| **Answer choices** | **Nonpractitioners**  Percentage No. | |
| --- | --- | --- |
| Yes | 14.3% | 4 |
| No | 82.1% | 23 |
| Not sure | 3.6% | 1 |
| Total | 100.0% | 28 |

Multiple choice question. Answered by 28 non-practitioners.

Note:

Non-practitioners include radiation oncologists and physicists who do not practice SFRT; radiobiologists, molecular biologists or related scientists.

This data is limited by small numbers of responses (4 responses).

**Qu 57: What type of tumors do your clinical colleagues treat with SFRT?  Check all that apply**

| **Answer choices** | **Nonpractitioners**  Percentage No. | |
| --- | --- | --- |
| Not sure which diseases are treated | 33.3% | 1 |
| Metastases to lymph nodes | 0% | 0 |
| Metastases to bones | 0% | 0 |
| Metastases to intraabdominal structures | 33.3% | 1 |
| Metastases to brain | 0% | 0 |
| Metastases to lungs | 33.33% | 1 |
| Primary (non-metastatic) head and neck cancer | 33.3% | 1 |
| Primary (non-metastatic) sarcoma | 66.7% | 2 |
| Primary (non-metastatic) lung cancer | 66.7% | 2 |
| Primary (non-metastatic) cervical cancer | 33.3% | 1 |
| Primary (non-metastatic) prostate cancer | 0% | 0 |
| Primary (non-metastatic) breast cancer | 0% | 0 |
| Primary malignant brain tumors | 0% | 0 |
| Primary (non-metastatic) malignant melanoma | 0% | 0 |
| Total | 100% | 9 |

Check box question. Answered by 3 non-practitioners.

Note:

Data is limited by small number of responses (3 responses).

**Qu 58: Do your clinical colleagues use any of the following SFRT dose schedules for patients?  Check all that apply**

| **Answer choices** | **Nonpractitioners**  Percentage No. | |
| --- | --- | --- |
| Not sure what dose/fractionation | 33.3% | 1 |
| 15 Gy in 1 fraction | 0% | 0 |
| 18 Gy in 1 fraction | 0% | 0 |
| 20 Gy in 1 fraction | 0% | 0 |
| 24 Gy in 3 fractions | 0% | 0 |
| Other doses and/or fractionations. Please specify: | 66.7% | 2 |
| Total | 100% | 3 |

Check box question. Answered by 3 non-practitioners.

Note:

Data is limited by small number of responses (3 responses).

In aggregate, the small numbers of responses regarding clinical SFRT may suggest that awareness of SFRT is still low and education in clinical SFRT is insufficient.

**Qu 59: Have you or your colleagues observed that any of the following SFRT prescription parameters may influence local tumor control in clinical patients?**

| **Parameter** | **Yes** | **No** | **Not sure** | **Parameter not used** | **Did not respond*** | **Total** | **Weighted average** |
| --- | --- | --- | --- | --- | --- | --- | --- |
| SFRT prescription dose | 66.7% 2 | 33.3% 1 | 0% 0 | 0% 0 | 0% 0 | 3 | 1.3 |
| Number of SFRT fractions (1 vs more than 1) | 33.3% 1 | 0% 0 | 33.3% 1 | 33.3% 1 | 0% 0 | 3 | 2.7 |
| Mean dose | 0% 0 | 0% 0 | 33.3% 1 | 33.3% 1 | 33.3% 1 | 3 | 4.0 |
| Dose to GTV margin | 33.3% 1 | 0% 0 | 33.3% 1 | 0% 0 | 33.3% 1 | 3 | 3.0 |
| Valley dose (V) | 0% 0 | 0% 0 | 33.3% 1 | 33.3% 1 | 33.3% 1 | 3 | 4.0 |
| Peak dose (P) | 0% 0 | 33.3% 1 | 0% 0 | 33.3% 1 | 33.3% 1 | 3 | 3.7 |
| Valley/peak dose ratio (VPDR) or Peak/valley dose ratio (PVDR) | 0% 0 | 0% 0 | 33.3% 1 | 33.3% 1 | 33.3% 1 | 3 | 4.0 |
| Peak-to-peak distance | 0% 0 | 0% 0 | 0% 0 | 33.3% 1 | 66.7% 2 | 3 | 4.7 |
| Peak width (defined at 50% of the maximal dose) | 0% 0 | 0% 0 | 0% 0 | 33.3% 1 | 66.7% 2 | 3 | 4.7 |
| Dose covering 5% or 10% of PTV (D5 or D10) | 66.7% 2 | 0% 0 | 0% 0 | 0% 0 | 33.3% 1 | 3 | 2.3 |
| Dose covering 50% of PTV (D50) | 0% 0 | 0% 0 | 0% 0 | 33.3% 1 | 66.7% 2 | 3 | 4.7 |
| Dose covering 90% or 95% of PTV (D90 or D95) | 33.3% 1 | 0% 0 | 33.3% 1 | 0% 0 | 33.3% 1 | 3 | 3.0 |
| Volume receiving 5 Gy or receiving 8 Gy per fraction | 0% 0 | 0% 0 | 33.3% 1 | 33.3% 1 | 33.3% 1 | 3 | 4.0 |
| Volume to Vertices / Volume to GTV (ratio) (in Lattice therapy) | 0% 0 | 0% 0 | 33.3% 1 | 33.3% 1 | 33.3% 1 | 3 | 4.0 |
| Vertices diameter (in Lattice therapy) | 0% 0 | 0% 0 | 33.3% 1 | 33.3% 1 | 33.3% 1 | 3 | 4.0 |
| Vertices separation (in Lattice therapy) | 0% 0 | 0% 0 | 33.3% 1 | 33.3% 1 | 33.3% 1 | 3 | 4.0 |
| Number of vertices (in Lattice therapy) | 0% 0 | 0% 0 | 33.3% 1 | 33.3% 1 | 33.3% 1 | 3 | 4.0 |
| Conventional external beam radiation dose (given in addition to SFRT) | 0% 0 | 33.3% 1 | 33.3% 1 | 0% 0 | 33.3% 1 | 3 | 3.3 |
| Equivalent uniform dose (EUD) of the SFRT | 0% 0 | 33.3% 1 | 33.3% 1 | 0% 0 | 33.3% 1 | 3 | 3.3 |
| Timing of SFRT dose (before, during or after)conventional radiation (please specify below) | 0% 0 | 33.3% 1 | 33.3% 1 | 0% 0 | 33.3% 1 | 3 | 3.3 |

Matrix/rating scale question. The weighted average column was calculated after converting the categories to numbers from 1 (always) to 6 (did not respond), where the average was weighted by the number of responses with that category. Answered by 3 non-practitioners.

*Non-practitioners who responded to at least one item were included in the calculations.

Note:

Data is limited by small number of responses (3 responses).

Note:

I addition to the questions presented, questions 36, 49 and 60 solicited free-text comments from radiation oncologists, physicists and non-practitioners.
